# Three-dimensional pore characterization of intact loess and compacted loess with micron scale computed tomography and mercury intrusion porosimetry

**DOI:** 10.1038/s41598-020-65302-8

**Published:** 2020-05-22

**Authors:** Linxin Zhang, Shengwen Qi, Lina Ma, Songfeng Guo, Zhiqing Li, Guoliang Li, Jijin Yang, Yu Zou, Tonglu Li, Xiaokun Hou

**Affiliations:** 1 Key Laboratory of Shale Gas and Geoengineering, Institute of Geology and Geophysics, Chinese Academy of Sciences, Beijing, China; 2 Chinese Academy of Sciences, Beijing, China; 3 University of Chinese Academy of Sciences, Beijing, China; 4 Department of Geological Engineering, Chang’an University, Xi’an, Shaan’xi China

**Keywords:** Civil engineering, Solid Earth sciences

## Abstract

The pore structure is one of the most important properties of soil, which can directly affect the other properties such as water content, permeability and strength. It is of great significance to study the soil pore structure for agricultural cultivation, water and soil conservation and engineering construction. This paper investigates the 3D pore characterization of intact loess and four kinds of compacted loess (with different dry density) in northwest China. Micro scale computed tomography and mercury intrusion porosimetry tests were performed to get the porosity, specific surface area, pore size distribution, connected pores content and isolated pores content of different samples. Results show that the intact loess has more connected pores than the compacted loess, and the compacted loess whose dry density appears to be modelled well still have different pore structure with the intact loess. In addition, as the compactness increasing, the large pores (>13 μm) were firstly broken into medium pores (8~13 μm) and some small pores (<8 μm) until the pore structure was close to the natural structure of the intact loess, after that medium pores began to be broken into small pores.

## Introduction

Loess is a type of Quaternary aeolian silt that has a global distribution in semi-arid and arid regions. In China, loess mantles more than 6% of the territory and the well-known Loess Plateau occupies a coherent area of more than 300,000 km^2^^1^. Structural collapse and subsidence are two typical engineering geological problems in Loess Plateau area^2,3^. As loess has an open and metastable structure which has been thought of the basic condition to cause subsidence^4,5^, a large number of scholars have carried out studies on the microstructure characteristics of loess.

Since the middle of last century, scholars have started to use optical microscopy to investigate the structure of loess pore. Due to technical limitations, the pore size observed at that time was generally more than 100 microns. Zhang^6^ observed the microstructure of intact loess in Gansu province of China under polarizing microscope, and found that the microstructure of loess was closely related to its engineering geological properties. By observing intact loess samples from different regions, the macropores were found to be irregular in shape and variable in size^7,8^.

Since the 1970s, the use of scanning electron microscopy (SEM) gradually led to advances in quantitative examination of loess pores. Due to its high precision, the researchers can observe the microscopic particle morphology and pore structure of loess samples on the micron or even submicron scales. Using SEM, Klukanova and Sajgalik^9^ examined the loess fabric caused by collapse and pointed out that the microstructure and porosity had certain effects on the collapsibility of loess. By analyzing the SEM images, it can be easily to get some 2D pore parameters, such as the equivalent radius, length, aspect ratio and so on^10^. Wang, *et al*.^11^ statistically analyzed the void area ratio of intact loess samples with different water content, and indicated that the void area ratio in the microphotograph decreased as the initial water content increased. Jiang, *et al*.^12^ took SEM observations on the natural and remolded samples before and after CU triaxial tests and found that the pores became disconnected and smaller with the confining pressure increased. And the pores in the natural sample were larger than those in the remold sample under the low confining pressure, while the pore size and its distribution were nearly identical for the natural and remolded samples when the confining pressure was up to 600 kPa as most cementation bonds in the natural sample had been broken. Xie, *et al*.^13^ analyzed documented changes in particles and micropore structure of loess-like soil during triaxial creep tests with SEM, and pointed out that mesopores which covered the largest area were the most important factors leading to loess-like soil creep.

Since the 1980s, mercury intrusion porosimetry (MIP) has become another quantitative analysis method to study the microstructure of loess, especially the porosity and the pore size distribution (PSD)^14^. Lei^4^ studied the PSD characteristics of the loess in China based on mercury intrusion porosimetry (MIP) tests, and classified loess pores into four types according to the size: the large pores (>0.016 mm in radius), the medium pores (0.016–0.004 mm), the small pores (0.004–0.001 mm) and the micropores (radius <0.001 mm). And the results have shown that the content of the medium pores of the samples after slumping on wetting decreased more than that of the original one, which indicated that the medium pores are important spaces for loess subsidence on wetting. By combining MIP and SEM, quantitative representation of the loess pore microstructure has become more accurate^12,15,16^.

However, all the images obtained by SEM are 2D images and the results are highly depend on the choice of observing directions, which will result in non-unique shape descriptors for pores and particles^17^. To reflect the truly geometric features of the pore networks, Wei, *et al*.^14^ generated serial SEM images by looping the polishing and image acquisition, and reconstructed the 3D loess microstructure. In consideration of maintaining the original structure of the sample to the maximum extent during the thickness removal, the loess pores were filled with the epoxy resin mixture^18^. In this case, it is difficult to ensure that all microscopic pore structures remain in their original state. Analogously, because of the principle that forcing a non-wetting liquid (often mercury) to intrude into a material at high pressure to get the PSD, MIP is destructive and the results may not true^19,20^.

With the development of observation technology, researchers attempted to use computed tomography (CT) to obtain the 3D pore structure of soil in recent years^21,22^. CT scan can be used to see the images of each layer inside the sample without interfering with the test process, and the image data can be analyzed mathematically to obtain the required information. Pu, *et al*.^23^ took the lead in applying CT (whose resolution was close to 1 mm) to the observation of the internal structure of undisturbed loess and observed that the macro-behavior of sample is closely related with its microstructure change. With CT and MATLAB, Li, *et al*.^24^ constructed the 3D macropore models of intact Malan loess at a resolution of 59 μm, and the results indicated that the pore connectivity in the vertical direction was better than that in the horizontal direction. With a resolution of 1 μm, Wei, *et al*.^25^ investigated the 3D pore network characterization of intact loess and paleosol samples in the South Jingyang Plateau, and it was further clarified that clay content could obviously affect the pore structure.

Previous studies have shown that the original macropore structure of loess can be destroyed into small pores under the action of high external force^1,10,12^. Moreover, loess with relatively small pores is more stable than that with large pores^4,26^. Thus, people generally compact the loess to increase its stability in actual engineering project^27–29^. However, a large number of studies have shown that despite the compaction measures, the remolded loess still has collapsibility which can be even more serious than the original loess^30–33^. Therefore, it is really necessary to study the microstructure of compacted loess. Using MIP tests, Rendell^34^ compared the PSD of naturally consolidated loess and laboratory compacted loess, and found that the pore distribution of artificially compacted loess was different from that of naturally consolidated loess. Wu, *et al*.^35^ analyzed the microstructure characteristics of compacted loess by SEM and divided the pores of compacted loess into two categories, scaffold pores and mosaic pores. Wang, *et al*.^36^ did a comparative study on the evolution of pore-size distribution of intact loess and remolded loess due to consolidation by SEM and MIP tests. The results have shown that remolded loess has very different microstructure and PSD from intact loess even though they may have the same grain-size distribution (GSD), mineralogical composition, and some other physical properties

In sum, the comparative study between intact loess and compacted loess from the aspect of 3D geometrical pore microstructure at high resolution, especially based on the non-destructive CT scanning technology, is very limited. Quantitative analysis of the microscopic pores of compacted loess samples under undisturbed conditions is urgently needed.

In this paper, quantitative analysis and comparison were carried out to characterize the 3D pore microstructure of the intact and compacted loess from the Loess Plateau. At the same time, the differences between the MIP test results and the high-resolution scan results of the same sample were compared and analyzed. This research is helpful for further understanding the 3D pore geometrical characteristics of intact and compacted loess and provides insights into the study of mechanical mechanism of loess behavior.

## Results

The 3D quantitative parameters of the pore structure were extracted for each of the loess samples, including porosity, specific surface area, PSD, connected pores content and isolated pores content. The definition of the parameters and how to get them were explained in the Methodology. The results were analyzed as described below.

### Porosity

Porosity is a single-value quantification of the amount of space available to fluid within a specific body of soil and can reflect the compactness of soil. Table 1 shows the porosity of the samples from different tests. As soil samples with dry density of 1.50 g/cm^3^ were relatively loose, the small size YA-l6-1.5 sample was damaged during removal from the plastic tube. Thus, there was no n_m1_ for YA-l6-1.5. It can be easy to find that the porosity from CT scanning (n_0_) is different from the porosity from MIP test (n_m_). And for results of the MIP tests, the porosity of small size samples (n_m1_) is greater than that of large size samples (n_m2_). Although the sample used in CT scanning and that used in the small-size MIP tests were the same one, the results from CT scanning were more similar to those from the large size MIP tests, and the results from the two experiments on intact loess were the most similar. By comparing the results of YA-L6-1.5, YA-L6-1.6, YA-L6-1.7 and YA-L6-1.8, it can be found that the porosity of the compacted loess samples decreases as the dry density increases. And from CT scanning results, the intact loess has greater porosity than the compacted loess, even though YA-L6-1.5 has a smaller dry density.

**Table 1:** Porosity of the samples from different tests.

Sample	Porosity from CT scanning n_0_(%)	Porosity from MIP tests		$\frac{{{\boldsymbol{n}}}_{{\boldsymbol{m}}{\bf{1}}}{\boldsymbol{-}}{{\boldsymbol{n}}}_{{\bf{0}}}}{{{\boldsymbol{n}}}_{{\boldsymbol{m}}{\bf{1}}}}$	$\frac{{{\boldsymbol{n}}}_{{\boldsymbol{m}}{\bf{2}}}{\boldsymbol{-}}{{\boldsymbol{n}}}_{{\bf{0}}}}{{{\boldsymbol{n}}}_{{\boldsymbol{m}}{\bf{2}}}}$
		Small size n_m1_(%)	Large size n_m2_(%)		
YA-L6-Intact	34.3030	64.9128	34.7519	0.47	0.01
YA-L6-1.5	34.1537	/	46.8373	/	0.27
YA-L6-1.6	27.0627	39.2095	31.3325	0.31	0.14
YA-L6-1.7	26.0750	48.5640	38.2527	0.46	0.32
YA-L6-1.8	25.6171	47.6530	29.8983	0.46	0.14

${n}_{0}$—the ratio of the number of pore voxels to the number of all voxels from CT scanning.

${n}_{m1}$—the ratio of total pore volume (cm^3^) to the bulk sample volume (cm^3^) of the small size sample from MIP test.

${n}_{m2}$—the ratio of total pore volume (cm^3^) to the bulk sample volume (cm^3^) of the large size sample from MIP test.

### Specific surface area

Specific surface area is one of the important parameters for studying soil pore structure and matric suction. Table 2 shows the specific surface area for different samples from different tests. As the micro-CT resolution is 1 μm, the specific surface area from CT scanning (S_0_) only considered the pores whose equivalent pore diameters are larger than 1 μm.

**Table 2:** Specific surface area for different samples from different tests.

Sample	Specific surface area from CT scanning S_0_ (μm^2^/μm^3^)	Specific surface area from MIP tests	
		All the pores S_M_ (μm^2^/μm^3^)	The pores (d > 1 μm) S_L_ (μm^2^/μm^3^)
YA-L6-Intact	0.2647	4.0732	0.2035
YA-L6-1.5	0.2267	7.0238	0.1919
YA-L6-1.6	0.2009	9.4047	0.1699
YA-L6-1.7	0.2005	7.0453	0.1504
YA-L6-1.8	0.2007	8.3930	0.1574

${S}_{0}$—the area of the total pore boundary divided by the volume of the soil skeleton from CT scanning.

${S}_{M}$—the total surface area of all the pores divided by the particle skeleton volume of the large size sample from MIP test.

${S}_{L}$—the total surface area of pores (d > 1 μm) divided by the particle skeleton volume of the large size sample from MIP test.

For results from CT scanning, it can be easy to find that the intact loess has a larger specific surface area (S_0_) than the compacted loess. For the compacted loess samples, as dry density increasing, S_0_ has become smaller and gradually approached a constant value, 0.20 μm^2^/μm^3^. The MIP tests have measured a completely different result, that the compacted loess has a larger specific surface area (S_M_) than the intact loess. And for the same sample, S_M_ can be tens of times as much as S_0_. However, only considering pores whose diameter are larger than 1 μm, the results from MIP test (S_L_) are similar to those from CT scanning. By comparing S_M_ and S_L_, one can find that there are more pores whose diameter are less than 1 μm in the compacted loess than in the intact loess.

### Connected and isolated pores

The connected pores have constituted the main channel through which air and water flow in the loess soil. Table 3 shows the content of connected *(Nc)* and isolated pores (*Ni*) for different samples from CT scanning. It can be found that at a resolution of 1 μm, the ratio of connected pores to all the pores is really high. And the intact loess has more connected pores and less isolated pores than the compacted loess. As dry density increasing, the proportion of isolated loess has become higher and gradually approached a constant value, 0.30%, even though the proportion of connected pores is still decreasing.

**Table 3:** Nc and Ni for different samples from CT scanning.

Sample	Nc (%)	Ni (%)	$\frac{{\boldsymbol{N}}{\boldsymbol{c}}}{{\boldsymbol{N}}{\boldsymbol{c}}{\boldsymbol{+}}{\boldsymbol{N}}{\boldsymbol{i}}}$
YA-L6-Intact	34.22	0.08	0.9975
YA-L6-1.5	33.91	0.24	0.9928
YA-L6-1.6	26.75	0.31	0.9885
YA-L6-1.7	25.76	0.31	0.9879
YA-L6-1.8	25.32	0.30	0.9884

${N}_{c}$—the percentage of connected pores (%).

${N}_{i}$—the percentage of isolated pores (%).

### PSD

**Figure 1:**
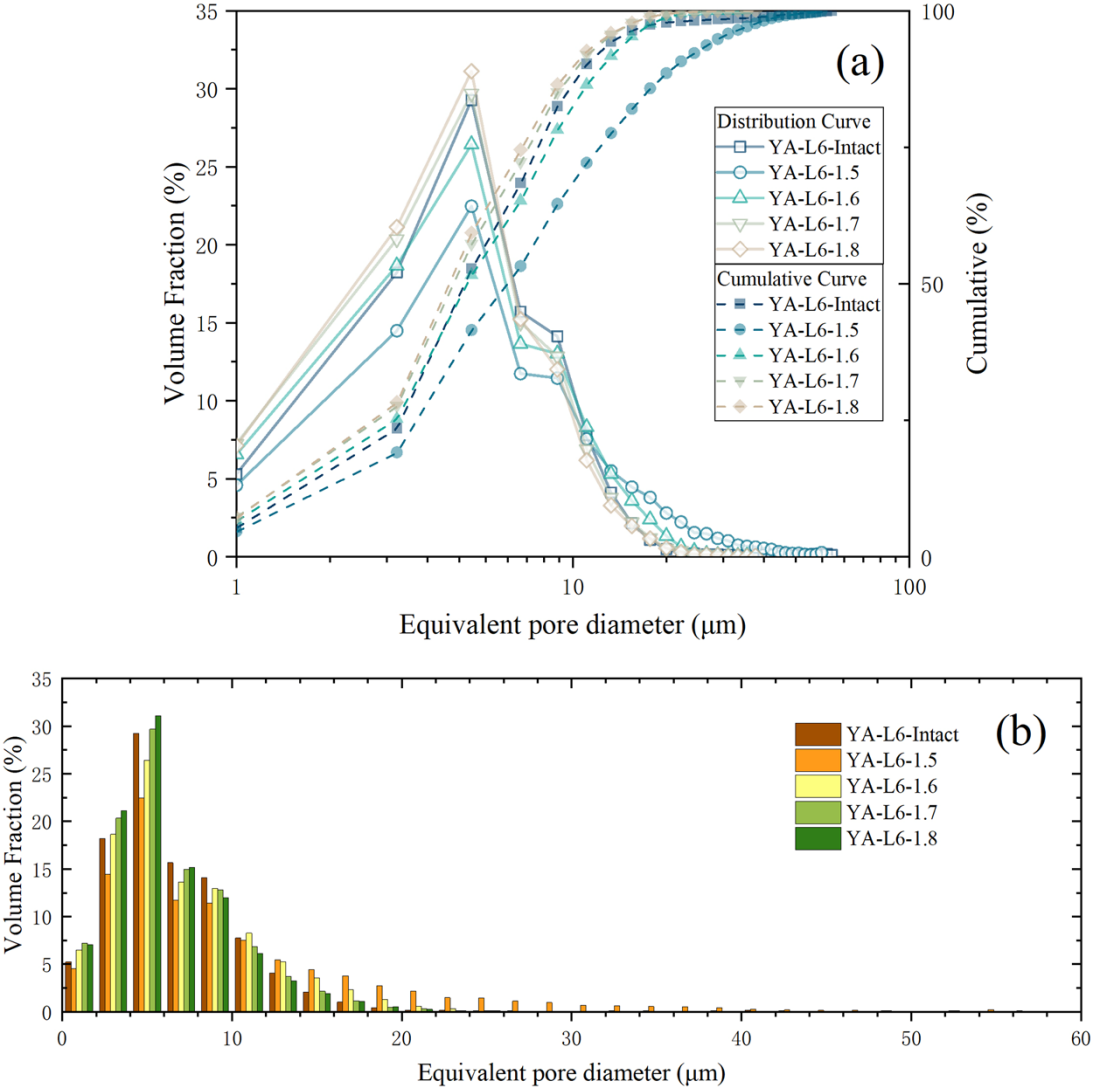
PSD of the five samples obtained from CT scanning: **(a)** line chart; **(b)** column chart.

The PSD can indicate complex pore structure characteristics in far more detail than porosity alone^37^. In this study, the micro-CT resolution is 1 μm, so the pores whose equivalent pore diameters are smaller than 1 μm cannot be detected. And as the volume of the reconstructed 3D structure is limited, there are no pores with an equivalent pore diameter larger than 60 μm. It can be found that the trend of PSDs is similar (Fig. 1). For the compacted loess samples, there are more large pores if the dry density is small. And the PSD of the intact loess sample YA-L6-Intact is more similar to those of the compacted loess samples with larger dry density, even though its dry density is closer to that of YA-L6-1.5.

**Figure 2:**
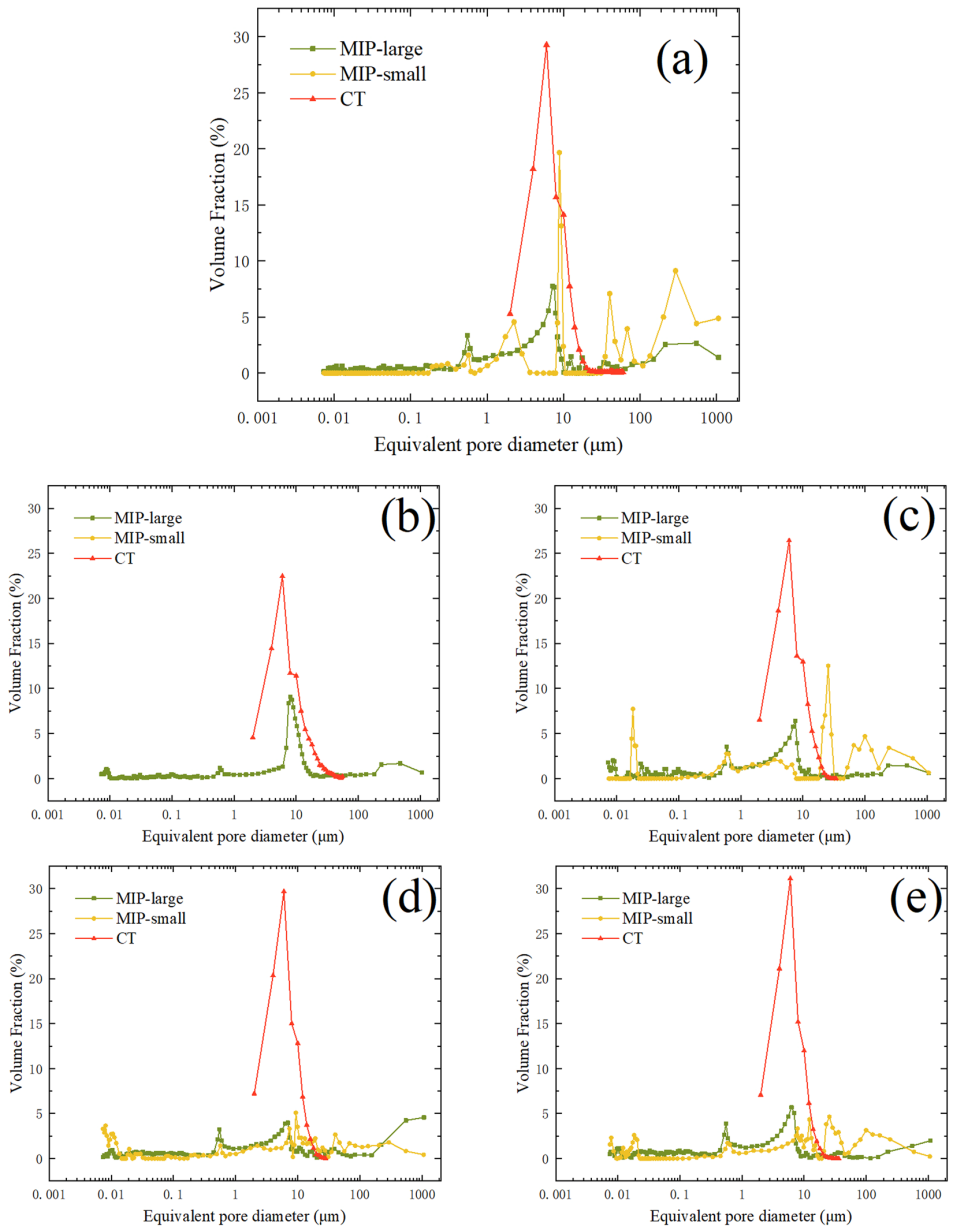
PSDs of the samples obtained from different tests: **(a)** YA-L6-Intact; **(b)** YA-L6-1.5; **(c)** YA-L6-1.6; **(d)** YA-L6-1.7; **(e)** YA-L6-1.8.

By comparing the small-size MIP tests and the large-size MIP tests (Fig. 2) one can find that the MIP-small curve is multi peaked and the MIP-large curve has two peaks at 0.6 μm and 8 μm, approximately. The results of the MIP tests also show that each sample has pores whose equivalent pore diameters are larger than 100 μm. And these large pores do not decrease with the dry density increasing. Except YA-L6-1.5, the MIP-large curves of different samples have the similar form. Moreover, comparing the results from the MIP tests and CT scanning (Fig. 2) the volume fractions under different equivalent pore diameters from the large-size MIP tests are less than 10% while those from the CT scanning can up to 30%. The PSDs from CT scanning are more concentrated and the CT curve has only one peak. From Fig. 1 one can find that the peak is concentrated in 4~6 μm.

**Figure 3:**
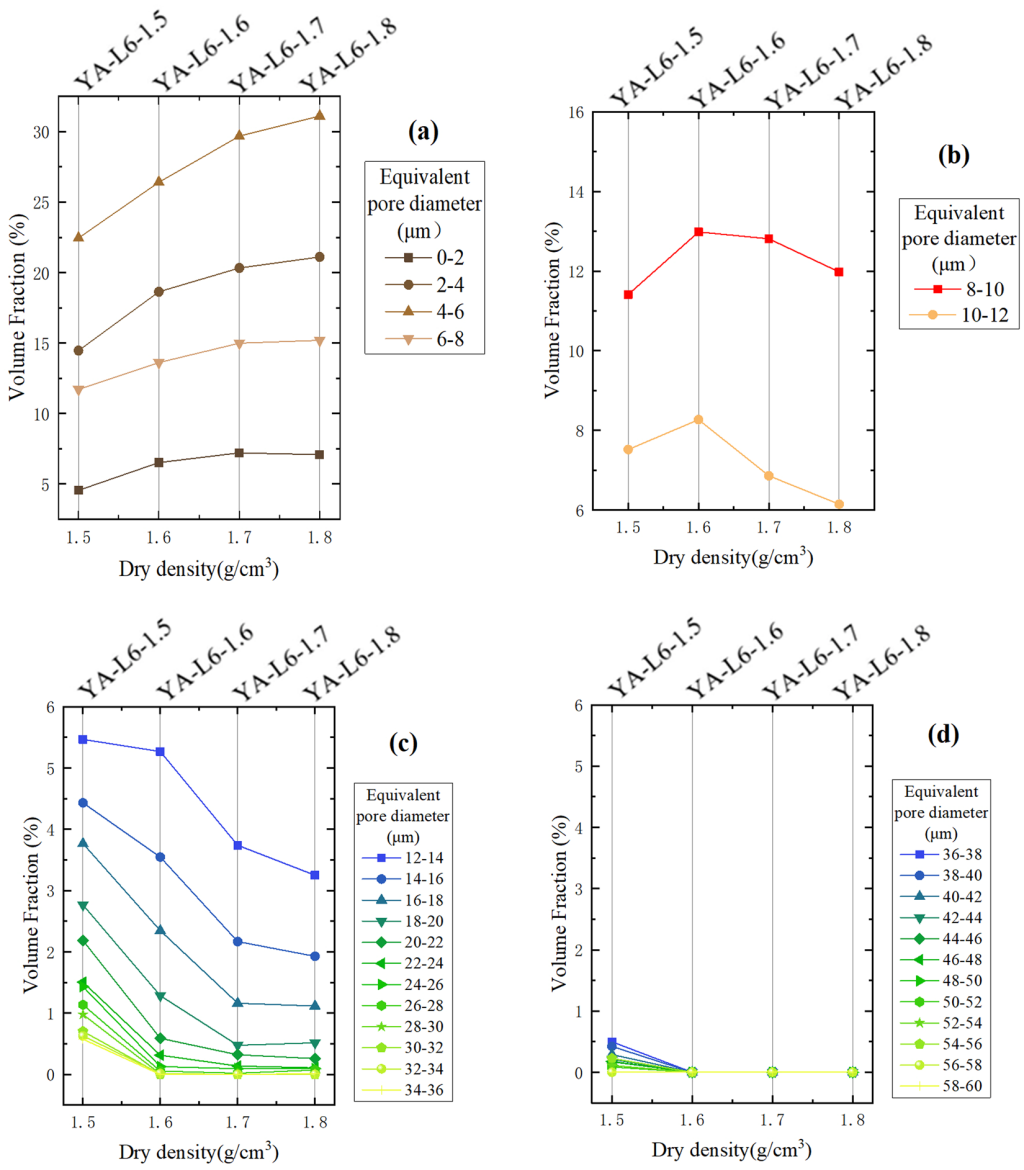
Volume fraction obtained from CT scanning for different equivalent pore diameter vs. dry density of the compacted loess samples: **(a)** pores with an equivalent diameter of 0~8 μm; **(b)** pores with an equivalent diameter of 8~12 μm; **(c)** pores with an equivalent diameter of 12~36 μm; **(d)** pores with an equivalent diameter larger than 36 μm.

From Fig. 3 one can find that the volume fraction of compacted loess samples under different equivalent pore diameters varies with dry density. For pores with an equivalent diameter of 0~8 μm, the volume fraction increases with the increasing of dry density (Fig. 3a). For pores with an equivalent diameter of 8~12 μm, the volume fraction increases first and then decreases with the increasing of dry density (Fig. 3b). For pores with an equivalent diameter of 12~14 μm, the volume fraction decreases slowly in the case of low compactness, then gradually decreases with the increasing of dry density. For pores with an equivalent diameter of 14~36 μm, the volume fraction gradually decreases with the increasing of dry density, and the decreasing extent reduces gradually (Fig. 3c). For pores with an equivalent diameter larger than 36 μm, the volume fractions are no more than 0.5%. Although the volume fraction also decreases with the increasing of dry density, it is relatively stable and the change is not obvious (Fig. 3d).

**Figure 4:**
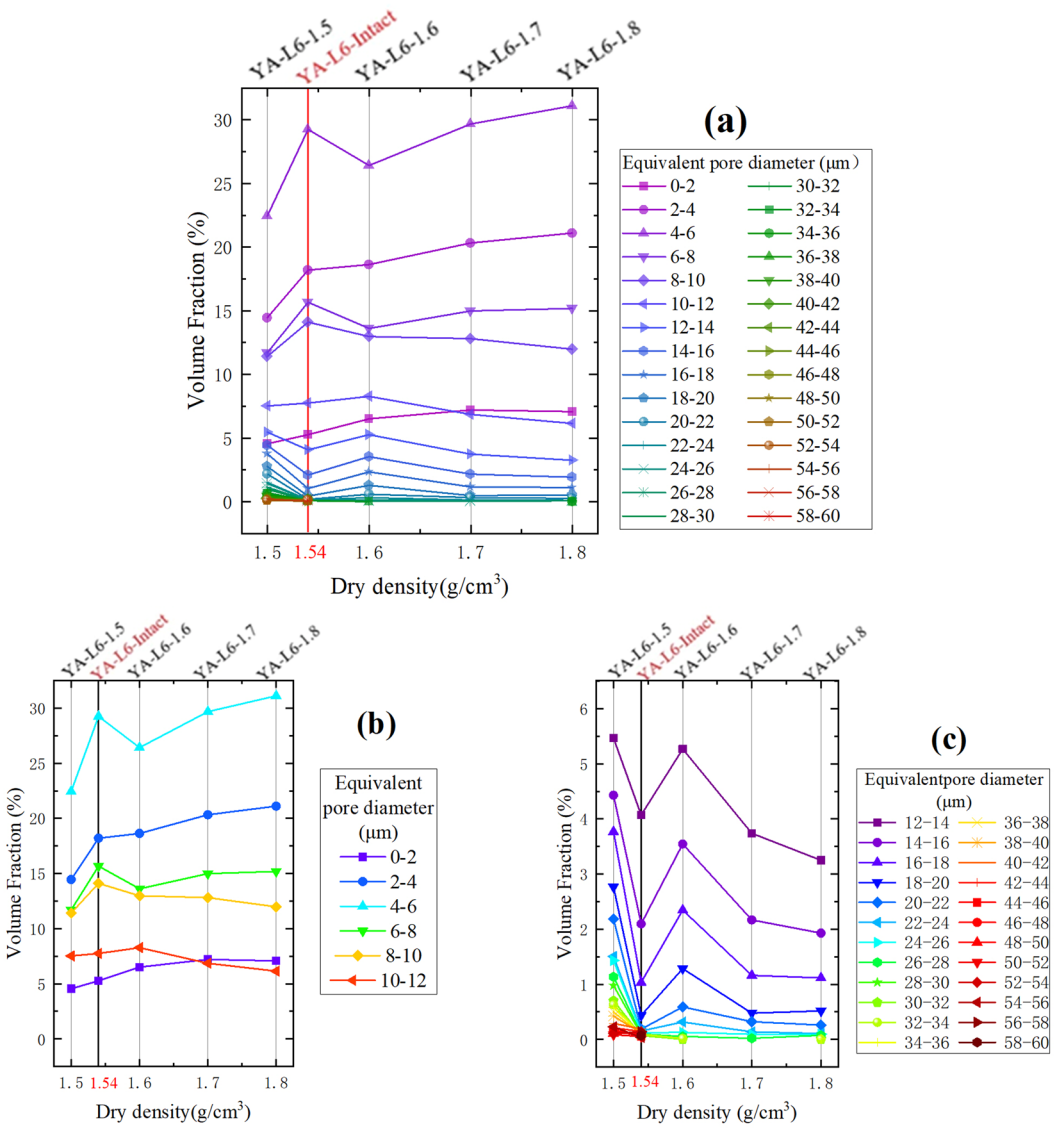
Volume fraction obtained from CT scanning for different equivalent pore diameter vs. dry density of the intact and compacted loess samples: (**a**) pores with an equivalent diameter of 0~60 μm; (**b**) pores with an equivalent diameter of 0~12 μm shown in (**a**); (**c**) pores with an equivalent diameter of 12~60 μm shown in (**a**).

Figure 4 shows the volume fraction of intact loess and four kinds of compacted loess samples under different equivalent pore diameters. It is obvious that the PSD of the intact loess sample is more similar to those of the compacted loess samples with larger dry density (Fig. 4a). Except the pores whose equivalent diameters are 0~4 μm and 8~10 μm, the volume fraction of other pores is just like that of YA-L6-1.7. Although the dry density of the intact loess is closer to that of YA-L6-1.5, the PSDs of the two samples are quite different. For pores with an equivalent diameter less than 10 μm, the volume fraction of the intact loess is higher than that of YA-L6-1.5 (Fig. 4b). And for pores with an equivalent diameter of 12~50 μm, the volume fraction of the intact loess is less than that of YA-L6-1.5 (Fig. 4c). However, for pores with an equivalent diameter of 10~12 μm, the volume fraction of the intact loess and two kinds of compacted loess (YA-L6-1.5 and YA-l6-1.6) are almost the same (Fig. 4b). And just like Fig. 3d, the difference of the pores whose equivalent diameters are larger than 50 μm between the intact loess and the compacted loess is not obvious (Fig. 4c).

## Discussion

### Comparison of CT technique and MIP

The afore mentioned results demonstrate that the porosity, specific surface area and pore distribution obtained from CT scanning are quite different from those obtained by MIP tests.

There are two main reasons to explain the differences in porosity (Table 1). Firstly, the CT scanning can only detect pores whose equivalent pore diameters are larger than 1 μm, while the MIP tests can detect pores with a diameter between 0.001 μm and 1000 μm. The direct difference between n_0_ and n_m_ is mainly from whether the pores, whose diameter are between 0.001 μm and 1 μm, were counted or not. This reason can also explain the differences in specific surface area (Table 2), that why S_M_ is greater than S_0_ and why S_L_ is more similar to S_0_. Secondly, the principle of MIP is to measure the volume of pores by the amount of mercury entering under different pressures. As there are many small bottlenecks inside the specimen, high-pressure intrusion of mercury may either deform or damage the soil structures, resulting in inaccurate estimates of soil porosity^17,19,20^. However, CT scanning is a nondestructive technique which will not interfere with or destruct the internal structure of soil, and the measurements of three-dimensional structures are based on actual observations rather than shape assumptions, so that it can accurately describe the true spatial characteristics of the pores^17,25^. Moreover, compared with large size samples, the small size samples are more likely to be disturbed during the test operation. For example, dried loess samples are prone to powder on the surface during manual sample preparation operation, and the powder volume of the small size sample accounts for a larger proportion of the total volume. And small size samples are more likely to be destroyed than the large size samples under the same pressure intrusion by mercury, due to the smaller external constraint volume of the pore. The above reasons can explain why n_m1_ is greater than n_m2_, and why n_m2_ is more similar to n_0_ (Table 1).

In this study, the PSDs from the CT scanning were obtained by counting the total volume of pores according to the range of equivalent diameter rather than the volume of each pore under a specific equivalent diameter. And the range is 2 μm. That’s one reason to explain why the volume fractions under different equivalent pore diameters from the CT scanning can up to 30%, which are much larger than the MIP results. Another reason is that some large pores with complex morphology were divided into many small pores according to the pore throat during parameter extraction. While in the results of MIP tests, these connected large pores were still independent pores with large equivalent diameters. Moreover, compared with MIP, X-ray CT can detect not only the connected pores but also isolated ones^20,38^. And this is the third reason to explain the large volume fractions. For the small pores with a diameter between 2 μm and 8 μm^4^, the peak of the MIP curve is concentrated in 8 μm while the peak of the CT curve is concentrated in 4~6 μm (see Figs. 1 and 2). This result indicates that the equivalent diameter of most isolated pores is 4~6 μm.

Although the CT technique does not interfere with or destruct the pore structure of the samples and produces a higher detectability of pores, there are still many limitations. Firstly, there is a conflict between the specimen size and the scanning resolution. Given a certain CT device, the specimen must be small enough to obtain high resolution images. However, small specimens cannot encompass macropores and the analysis of the macropores are not representative. In addition, pores with a diameter less than the resolution of the instrument cannot be detected. It is difficult to make small loess samples without artificial disturbance, and it cannot be fixed in experimental observation when the sample size is very small. Thus, MIP is still irreplaceable to study the nano pores of the loess.

### The differences of pore microstructure between intact loess and compacted loess

By analyzing the porosity of the intact and compacted loess obtained by different experiments, one can find that n_0_ and n_m2_ of the intact loess are basically the same, while there is a big difference between n_0_ and n_m2_ for the compacted loess (Table 1). By comparing the content of connected and isolated pores for different samples from CT scanning (Table 3), one can find that the intact loess has more connected pores than the compacted loess. Considering the discrepancies in porosity values detected by MIP and m-CT methods could help to determine the volume of closed or unconnected pores in the soils^38^, the results from Fig. 2 further confirm that. In addition, by comparing the specific surface area of the intact and compacted loess obtained by different experiments (Table 2), one can find that the compacted loess has more small pores (0.001 μm < d < 1 μm) than the intact loess. The results also suggest that the pore structure of the intact loess is more stable as a result of the long period of structural evolution of the loess, so that it cannot be easily changed under high pressure intrusion by mercury compared with the compacted loess. This structural evolution of the loess is closely related to the movement of water^24^. There is a certain amount of water in loess under natural condition, which mainly comes from precipitation, frost, and snow^39,40^. On one hand, the water seepage in loess facilitates the formation of connected pores in loess^41^. On the other hand, the infiltrating water dissolves soluble salts, causing transportation and reprecipitation of calcium carbonates^42^ and reinforcement of existing pores by cements^43^. However, the compacted loess has not experienced that structural evolution so that it contains more isolated pores and more small pores.

Another significant conclusion can be drawn from the data in Figs. 1 and 4, that the pore structure of the intact loess is more similar to that of the YA-L6-1.7, even though its dry density is closer to that of YA-L6-1.5. It suggests that the remolded loess in laboratory cannot represent the original loess with the same physical properties. In order to get similar pore structure, the remolded loess must be compacted until its dry density is slightly higher than that of intact loess. The result observed from the new method can further confirms the conclusion from Rendell^34^, that laboratory consolidated loess do not mirror those for naturally consolidated loess even though the density appears to be modelled well. And from Fig. 4, one can speculate that, when the dry density is identical, the compacted loess would have more small pores than the intact loess. Furthermore, from the data of pores whose equivalent diameter is 10~12 μm (Fig. 4b), there is a preliminary judgment that pores with a diameter of 10~12 μm cannot be easily destroyed under low compactness (i.e. dry density less than 1.6 g/cm^3^).

According to the changing trend of content with dry density increasing (Fig. 3), the pores can be divided into three categories base on the equivalent diameter: large pores (>13 μm), medium pores (8~13 μm) and small pores (<8 μm). It should be noted that the pores whose equivalent diameter is 12~14 μm, its content firstly decreases slowly. It means that the content of some small pores increases and that the content of some large pores decreases. For convenience, 13 μm is used as the dividing standard here. As the dry density increases, the content of small pores continues to increase while the content of large pores decreases rapidly first and then decreases slowly. However, the changing trend of medium pores content is first increasing and then decreasing. Results suggest that, with the continuous increasing of compactness, the large pores in the remolded loess were firstly broken into medium pores and some small pores until the pore structure was close to the natural structure of the intact loess. When the compactness was further increased, a lot of medium pores were broken into small pores. Analyzing Figs. 3 and 4, the volume fraction of pores (>13 μm) is stable for the intact loess and the compacted loess whose dry density is greater than its natural state. That may be due to the similar grain-size distribution (Table 4).

**Table 4:** Dry density and grain-size distribution of the samples.

Sample	Dry density *ρ* (g/cm^3^)	Grain-size distribution (%)		
		Clay(<2 μm)	Silt(2–50 μm)	Sand(>50 μm)
YA-L6-Intact	1.54	3.88	84.32	11.80
YA-L6-1.5	1.50	3.86	83.61	11.53
YA-L6-1.6	1.60	3.33	84.75	11.92
YA-L6-1.7	1.70	3.84	81.46	14.70
YA-L6-1.8	1.80	3.67	80.78	15.55

## Conclusions

In this paper, the three-dimensional pore structures of the intact and four kinds of compacted loess from the Loess Plateau were reconstructed based on micron scale computed tomography. By comparing porosity, specific surface area and PSD between the intact and compacted loess, and analyzing the results of CT scanning and MIP tests, the following important conclusions can be reached:

1. The intact loess has more connected pores than the compacted loess and the pore structure of the intact loess is much more stable.
2. The compacted loess cannot mirror the intact loess in pore structure even though the dry density appears to be modelled well.
3. The content of pores with a diameter of 10~12 μm is consistent with the natural state under low compactness.
4. The pores of the compacted loess are divided into three categories base on the equivalent diameter: large pores (>13 μm), medium pores (8~13 μm) and small pores (<8 μm). As the dry density increases, the large pores were firstly broken into medium pores and some small pores until the pore structure was close to the natural structure of the intact loess. When the compactness was further increased, a lot of medium pores were broken into small pores.

This paper did not carry out comparative study before and after the mechanical test, and the structure evolution of the intact and compacted loess under the stress state is still unknown. As a future work, comparative study before and after the mechanical test should be carried out to understand the collapse mechanism of the intact and compacted loess.

Furthermore, the method to study pore structure in this paper also provides a new idea for the study of soil structure in the fields of agricultural cultivation, water and soil conservation and engineering construction.

## Methodology

### Sampling and sample preparation

**Figure 5:**
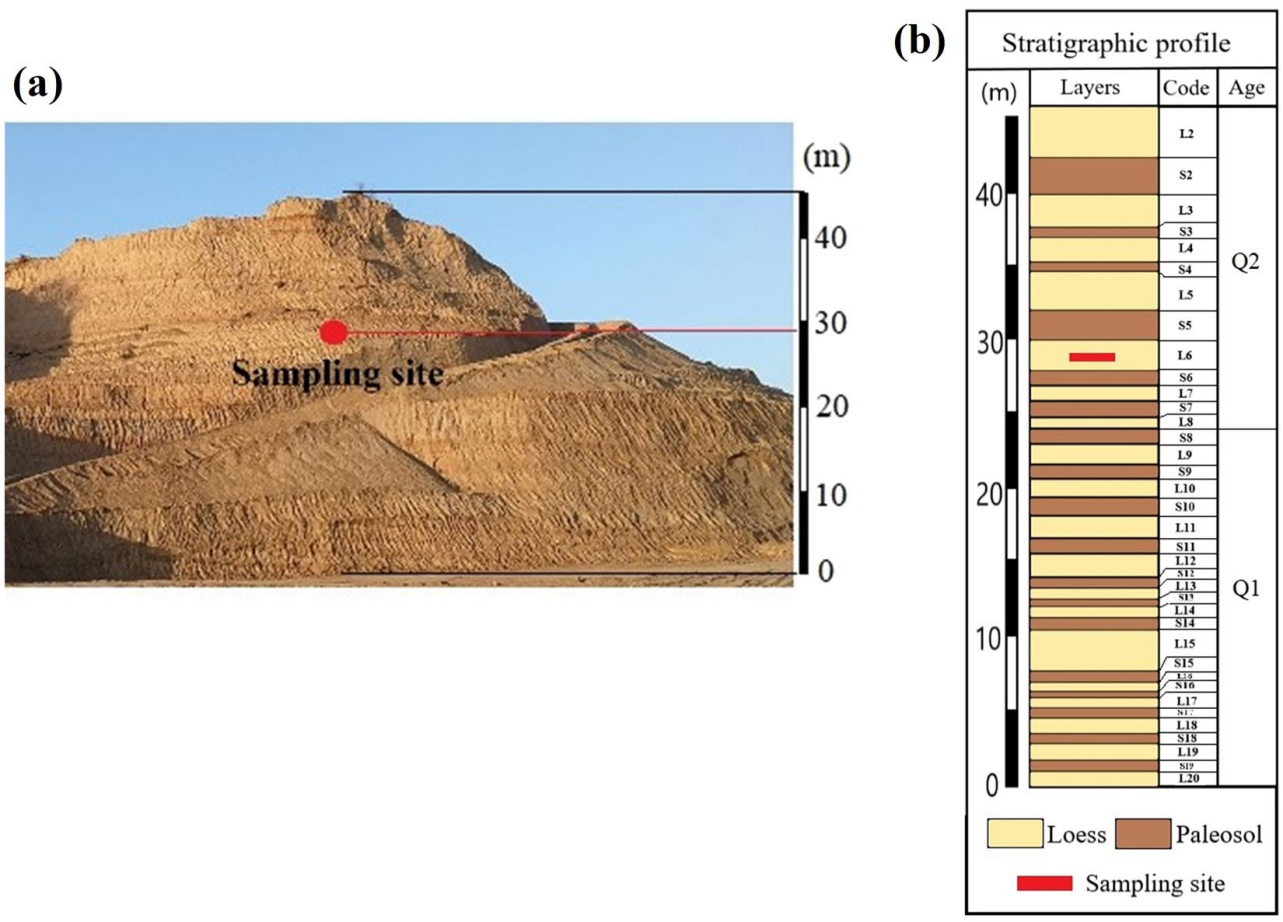
Location of the sampling site: (**a**) the sampling site in the excavated slope profile; (**b**) stratigraphic profile of the excavated slope.

The samples of loess used in this study were obtained from a slope in Yan’an City, Shannxi, China. The slope is about 46 meters high, and distinct loess strata can be observed (Fig. 5a). According to the study of loess strata by Liu^44^, a preliminary stratigraphic identification of the section was conducted in the field. Figure 5b shows the stratigraphic profile of the slope. The material composition and microstructure of loess in different strata are obviously different^4,24,25^. To ensure the uniformity, all the samples used in the tests all come from the same place in the same stratum, the L6 layer of Middle Pleistocene (Q2) strata.

To avoid disturbance during the transportation, the intact loess samples of cylindrical shape with diameter of 11 cm and height of 16 cm were well sealed by PVC pipe, plastic wrap and plastic tapes. Table 5 shows some physical properties of the randomly selected cylindrical loess samples, including the dry density, specific density, moisture content, liquid limit and plastic limit. All the parameters were determined according to Chinese standard for soil test method (GB/T 50123-1999). The results show that the physical properties of different cylinder samples are basically the same, which ensure that the compacted loess samples made from the other cylindrical samples have the similar specific density, liquid limit and plastic limit with the intact loess sample.

**Table 5:** Physical properties of the intact loess samples.

Sample	Dry density *ρ* (g/cm^3^)	Specific density *ρ* (g/cm^3^)	Moisture content *ω*/%	Liquid limit *W*_*L*_/%	Plastic limit *W*_*P*_/%
L6-01	1.53	2.70	12.7	28.4	18.9
L6-02	1.54	2.71	13.9	28.5	18.5
Average	1.54	2.71	13.3	28.5	18.7

The compacted loess used in the tests includes four different dry densities (1.5 g/cm^3^, 1.6 g/cm^3^, 1.7 g/cm^3^, 1.8 g/cm^3^) and the sample processing steps are as follows:

- The loose soil from intact cylindrical samples was selected and dried naturally. After fully crushed, it was passed through a 2 mm sieve and then was dried for no less than 8 hours at 100 °C.
- 1000 g dry soil and 100 g distilled water were mixed thoroughly and then sealed for more than 48 hours, to make sure that the moisture content of the compacted samples was 10%, as the grain profile of compacted loess under this water content was more clear^45^.
- Put the mental ring (61.8 mm × 20 mm) into the box of the sample making apparatus and then add the certain quality of soil according to the preset dry density.
- Put the box in the correct location in the sample making apparatus and apply pressure to the soil in the box by the jack at the bottom for more than 40 seconds.
- Take out the mental ring, cut the sample, weigh it and calculate the dry density of the processed sample. If the error was greater than 0.01 g/cm^3^ compared with the preset dry density, the sample would be discarded.

Table 4 shows the dry density, the clay(<2 μm), silt(2–50 μm) and sand(>50 μm) fractions of the samples^46^. The above data of particle size analysis were obtained from a Malvern Mastersizer3000 laser analyzer.

**Figure 6:**
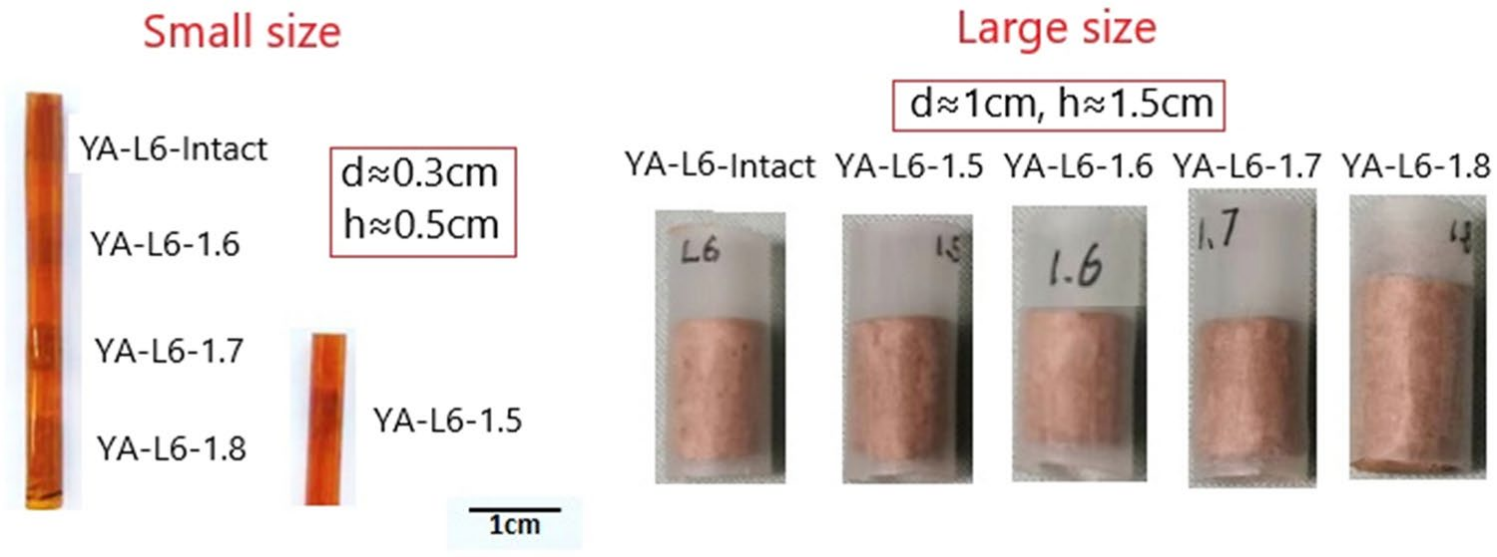
The small size and large size samples (small size samples for CT&MIP tests and large size for MIP test only).

As there was a lot of moisture in the initial samples, they were put in a dry place with constant temperature and ventilation for more than one month to meet the requirements of the CT scan and MIP tests. After air-drying, the samples were trimmed to cylindrical by extremely thin and sharp blades. For CT scan, the diameter of the samples was 3 mm and the height was 5 mm. For MIP tests, besides the small size samples, the diameter of the large size samples was 1 cm and the height was 1.5 cm. All the samples were encased in a plastic tube to prevent disturbance (Fig. 6).

### CT scanning

**Figure 7:**
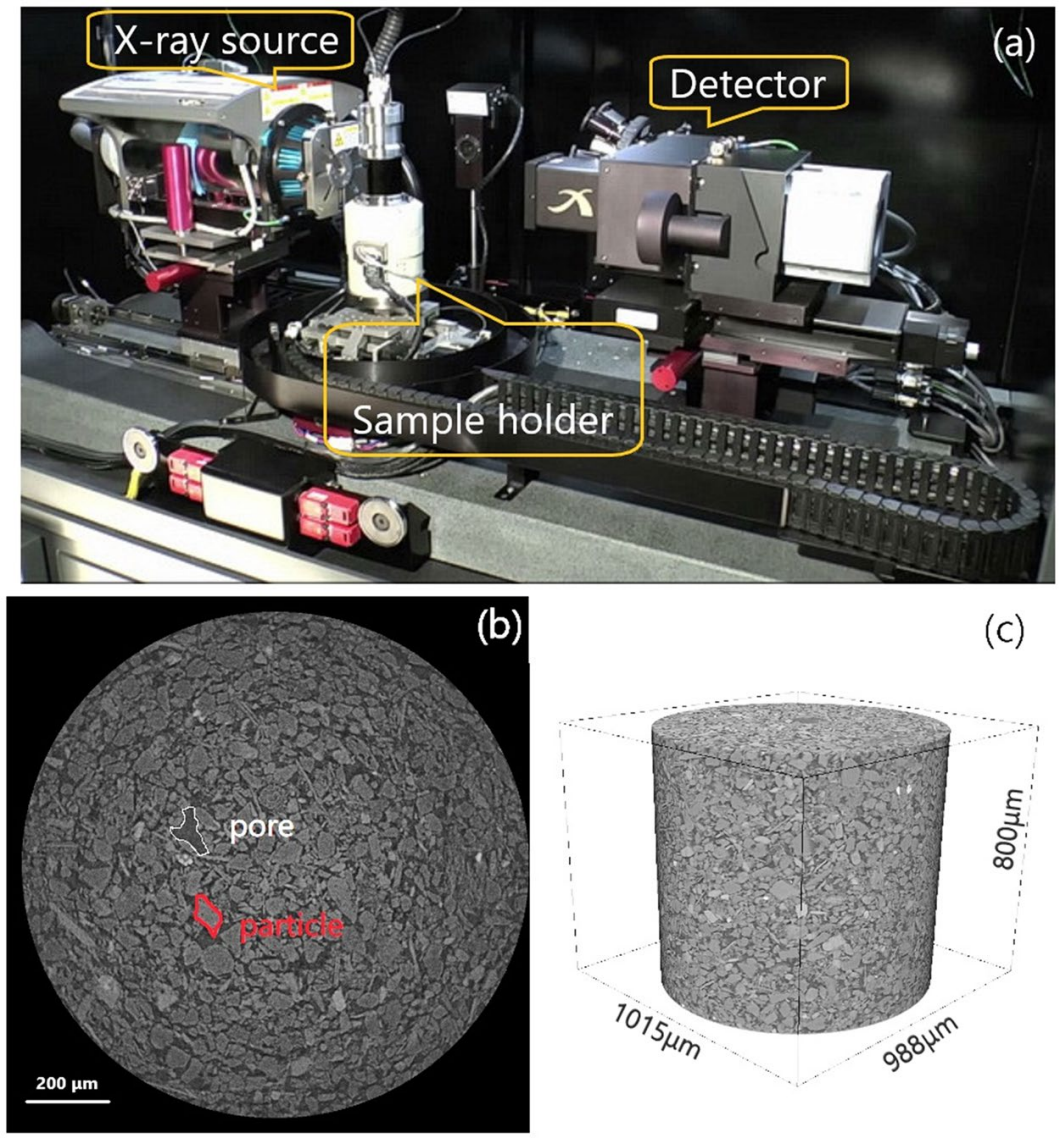
CT scan set-up: **(a)** the micro-CT scanner; **(b)** a grey scale image; **(c)** the cylindrical sample obtained from CT scanning.

A micro-CT device with high resolution (Xradia520 Versa from Zeiss) was used to scan the specimens, at the Institute of Geology and Geophysics, Chinese Academy of Sciences (Fig. 7a). The instrument can observe the three-dimensional appearance and internal structure of the sample without destroying the integrity of the sample, and realize the three-dimensional visualization of the observation results. The highest resolution (volume pixel) reached by the instrument is 0.35 micron. In this study, the scan system was set to 60 kV (voltage) and 81 μA (current), and the pixel size was 1 μm. After CT scanning, 997 gray images were obtained for each sample, and each image was consisted of 1,002,820 (1015 × 988) pixels with gray values in the range of 0–255. It can be easily to distinguish soil particles and pores by the difference of gray scale which represents the X-ray attenuation coefficient (Fig. 7b). The cylindrical sample can be obtained after the gray image combination (Fig. 7c). Table 6 shows the three-dimensional appearance of the scanned sample. The cylinder diameters obtained from scanning different samples were slightly different.

**Table 6:** The three-dimensional appearance of the scanned samples.

Sample	Cross section	Longitudinal section	3D structure
YA-L6-Intact			
YA-L6-1.5			
YA-L6-1.6			
YA-L6-1.7			
YA-L6-1.8			

### Data processing

**Figure 8:**
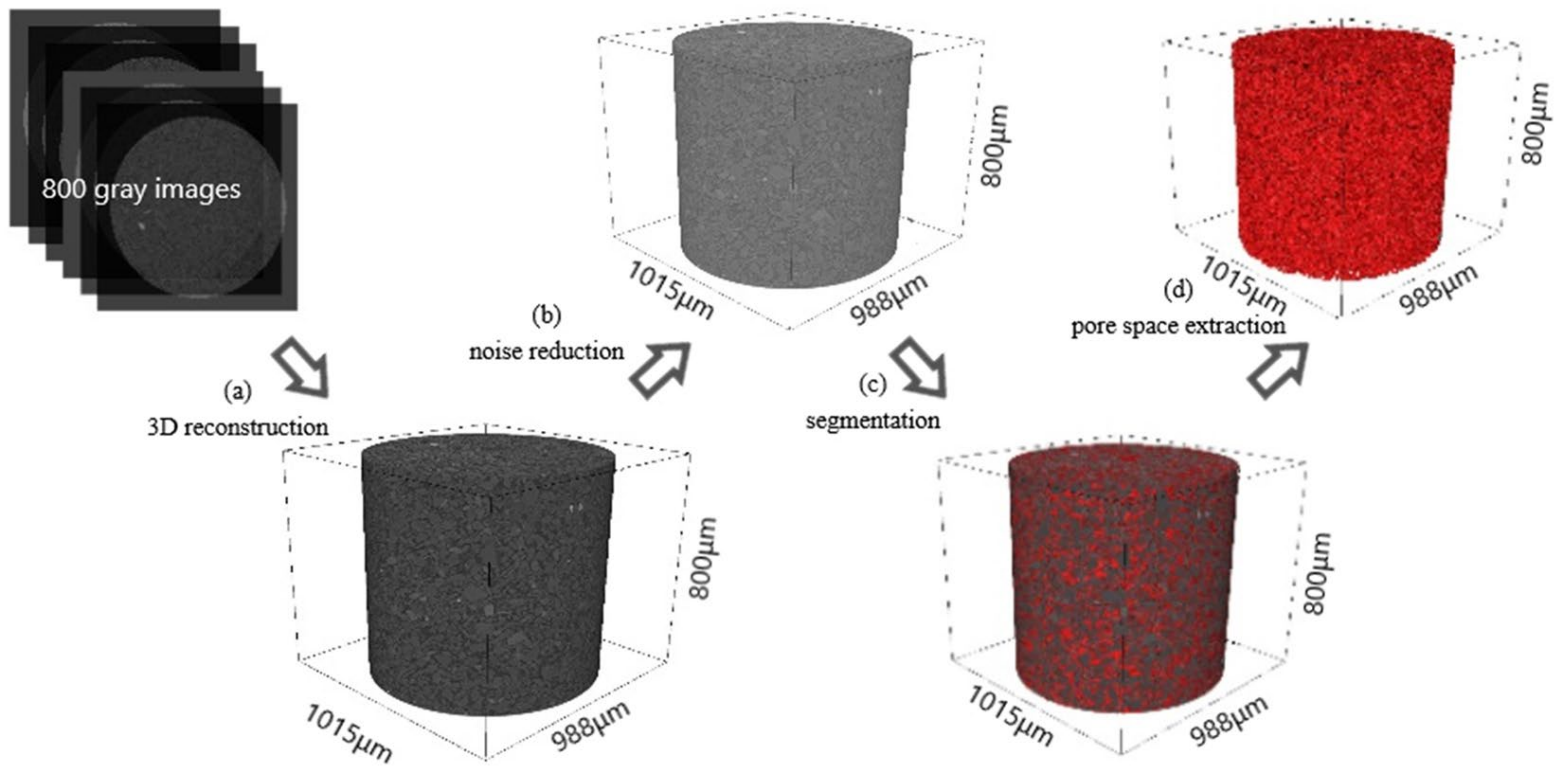
Workflow to reconstruct the 3D pore structure: **(a)** 3D reconstruction; **(b)** noise reduction; **(c)** segmentation; **(d)** pore space extraction.

The procedure to get the three-dimensional pore characterization consists of two main parts: 3D pore structure reconstruction and 3D parameter extraction. In addition, the 3D data can be easily visualized, analyzed and modeled with the Avizo software. Figure 8 shows the specific workflow to reconstruct the 3D pore structure.

Firstly, for each sample, 800 consecutive gray images were selected to load in Avizo to get the 3D microtomography volume (Fig. 8a). The 3D cylindrical volumes of the samples were 480,523,200 μm^3^ for YA-L6-Intact, 475,346,500 μm^3^ for YA-L6-1.5, 462,029,300 μm^3^ for YA-L6-1.6, 479,319,200 μm^3^ for YA-L6-1.7 and 450,771,200 μm^3^ for YA-L6-1.8, respectively. Secondly, to highlight image features before segmentation, the Gauss filter was used to reduce image noise or artifacts (Fig. 8b). Thirdly, particles and pores were segmented in a semi-automatic way by using interactive watershed tool and artificial selection, which could give more accurate results than commonly used segmentation method (Fig. 8c). Finally, the segmented pores were selected in 3D mode to generate new volume without the particle parts, and the 3D pore structure was obtained (Fig. 8d).

The 3D quantitative parameters, such as porosity, specific surface area and equivalent pore diameter, were extracted to depict the loess pore structure. Among them, the porosity and the specific surface area could be extracted directly with the analysis module in Avizo. Here, the porosity (n_0_) is defined as the ratio of the number of pore voxels to the number of all voxels (pore voxels and particle voxels) in the cylindrical volume. The specific surface area (S_0_) is defined as the area of the total pore boundary divided by the volume of the soil skeleton. In the meanwhile, the connected pores and isolated pores can be distinguished, and the voxel number of these two types of pores were also calculated. Here, Nc means the ratio of the number of connected pore voxels to the number of all voxels. Ni means the ratio of isolated pore voxels to the number of all voxels. According to Li and Yang^47,48^, the specific process of generating the equivalent pore diameter is as follows:

- A three-dimensional image corresponding to the sample to be measured was obtained and all pores in the three-dimensional image were extracted (Fig. 8).
- The pore throat identification system developed by Li and Yang^47^ of the Institute of Geology and Geophysics, Chinese Academy of Sciences, was used to segment the connected pores in the volume.
- On account of an isotropic voxel size was 1 μm in this study, the default geometry was set to a cube with a length of 2 μm.
- The number of cubes (2 μm × 2 μm × 2 μm) that could be filled at most in each pore was calculated, and the maximum number of fillings corresponding to each pore was obtained.
- The equivalent pore diameter corresponding to each pore was calculated according to the maximum number of fillings corresponding to each pore and the size of the geometry. By the way, the PSD was obtained.

### MIP test

In this study, mercury injection test was also carried out on the air-dried samples (both small size and large size) to statistics the PSD data, the porosity and the specific surface area in order to compare with the results from the CT scanning. The small size samples were the samples after CT scan. The MIP apparatus was the PoreMaster 60GT MIP device from Quantachrome Corporation.

Given the assumption of a cylindrical pore model and according to the definition of liquid surface tension, the relationship between the intrusion pressure *p* and the pore diameter *d*_*p*_ can be written as *d*_*p*_ = *−4γcosθ/p*, where *γ* is the surface tension of mercury at 20 °C (0.485 N/m) and *θ* is the contact angle between mercury and the solid phase (140°)^19,36,49–51^. The pore volume can be derived from the quantity of intruded mercury at the appropriate pressure step for the corresponding pore diameter.

Here, the porosity (n_m_) is defined as the ratio of total pore volume (cm^3^) to the bulk sample volume (cm^3^). The specific surface area (S_M_) is defined as the total surface area of all the pores divided by the particle skeleton volume of the large size sample from MIP test, and it can be calculated as the product of surface area (m^2^/g) and particle density (g/cm^3^). To compare with the results from CT scanning, the specific surface area (S_L_) of pores whose diameter are larger than 1 μm was also calculated. It is defined as the total surface area of pores (d > 1 μm) divided by the particle skeleton volume.

## Supplementary information

Supplementary Figure S1

SupplementaryFigure S2.

Supplementary Figure S3.

Supplementary Figure S4.

Supplementary Figure S5.

Supplementary Figure  S6.

Supplementary Figure S7.

Supplementary Figure S8.

Supplementary Figure S9.

Supplementary Figure S10.

Supplementary Figure S11.

Supplementary Figure S12.

Supplementary Figure S13.

Supplementary Figure S14.

Supplementary Figure S15.

Supplementary Figure S16.

## Data Availability

The data generated during and/or analyzed during the current study are available from the corresponding author on reasonable request.
