# Supplementary figures and images for: Three-dimensional pore characterization of intact loess and compacted loess with micron scale computed tomography and mercury intrusion porosimetry
Source: Sci Rep. 2020 May 22;10:8511. doi: 10.1038/s41598-020-65302-8 (PMC7244767; doi:10.1038/s41598-020-65302-8)

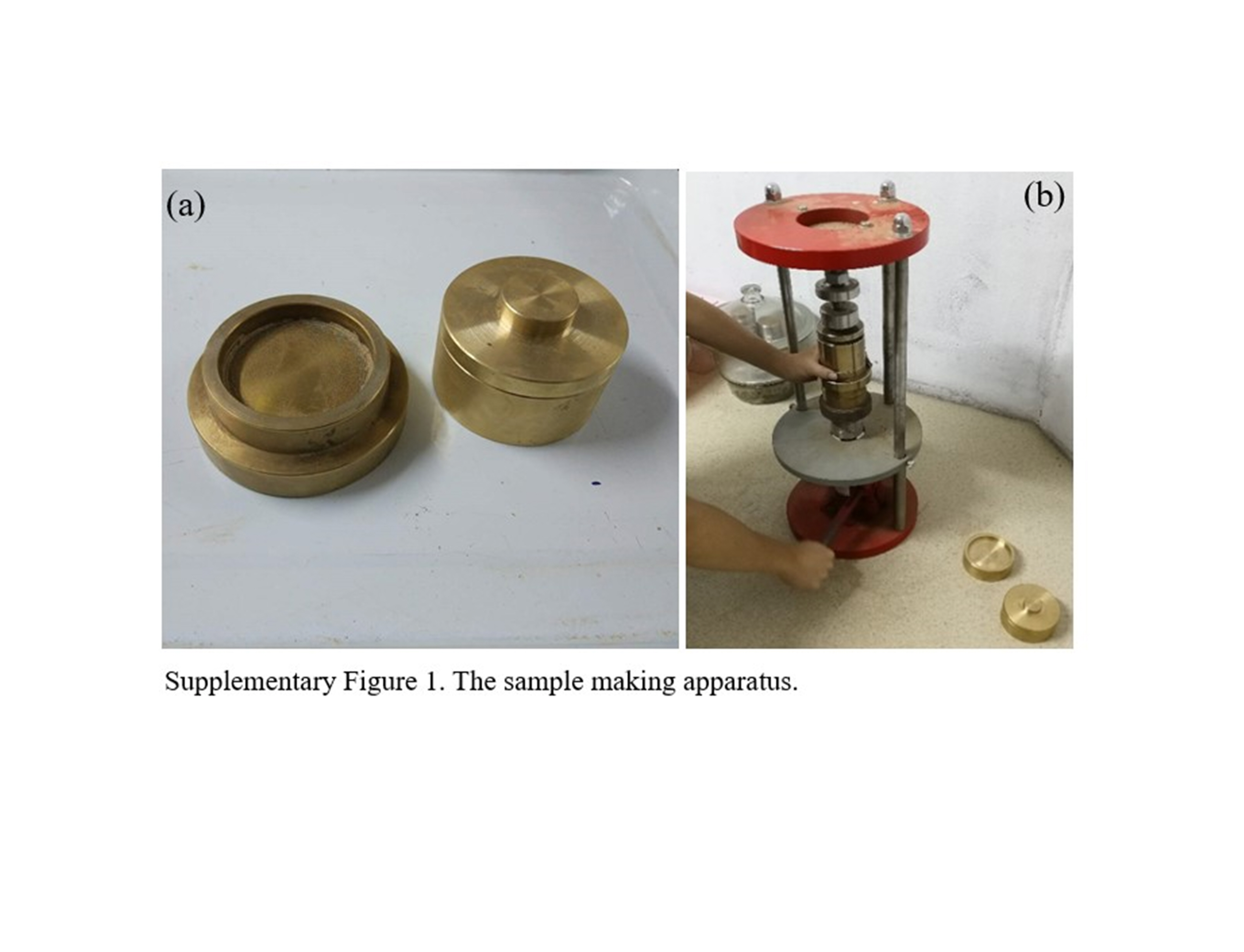

Supplement: Supplementary file 1 — Supplementary Figure S1 [file 41598_2020_65302_MOESM1_ESM.jpg]

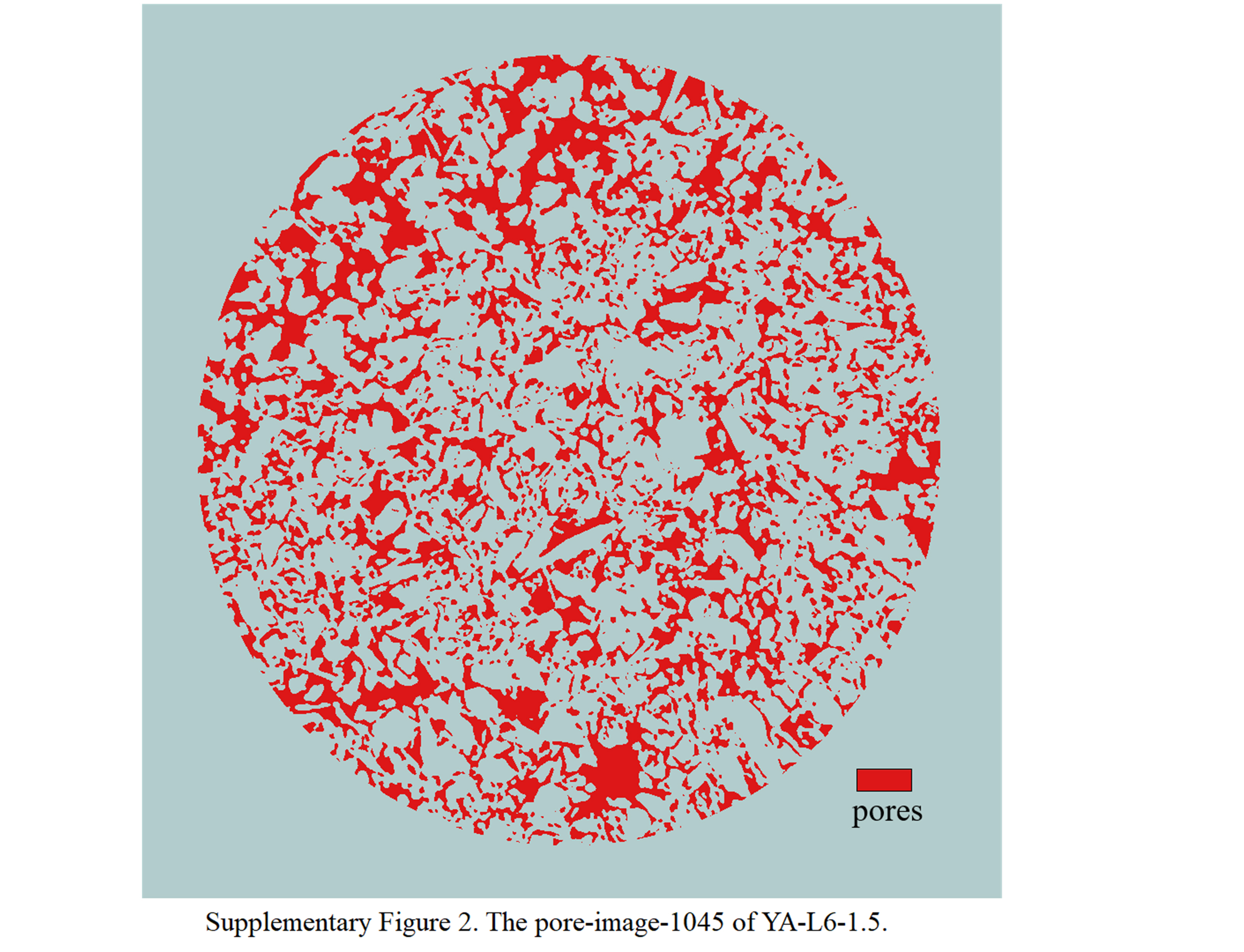

Supplement: Supplementary file 2 — SupplementaryFigure S2. [file 41598_2020_65302_MOESM2_ESM.jpg]

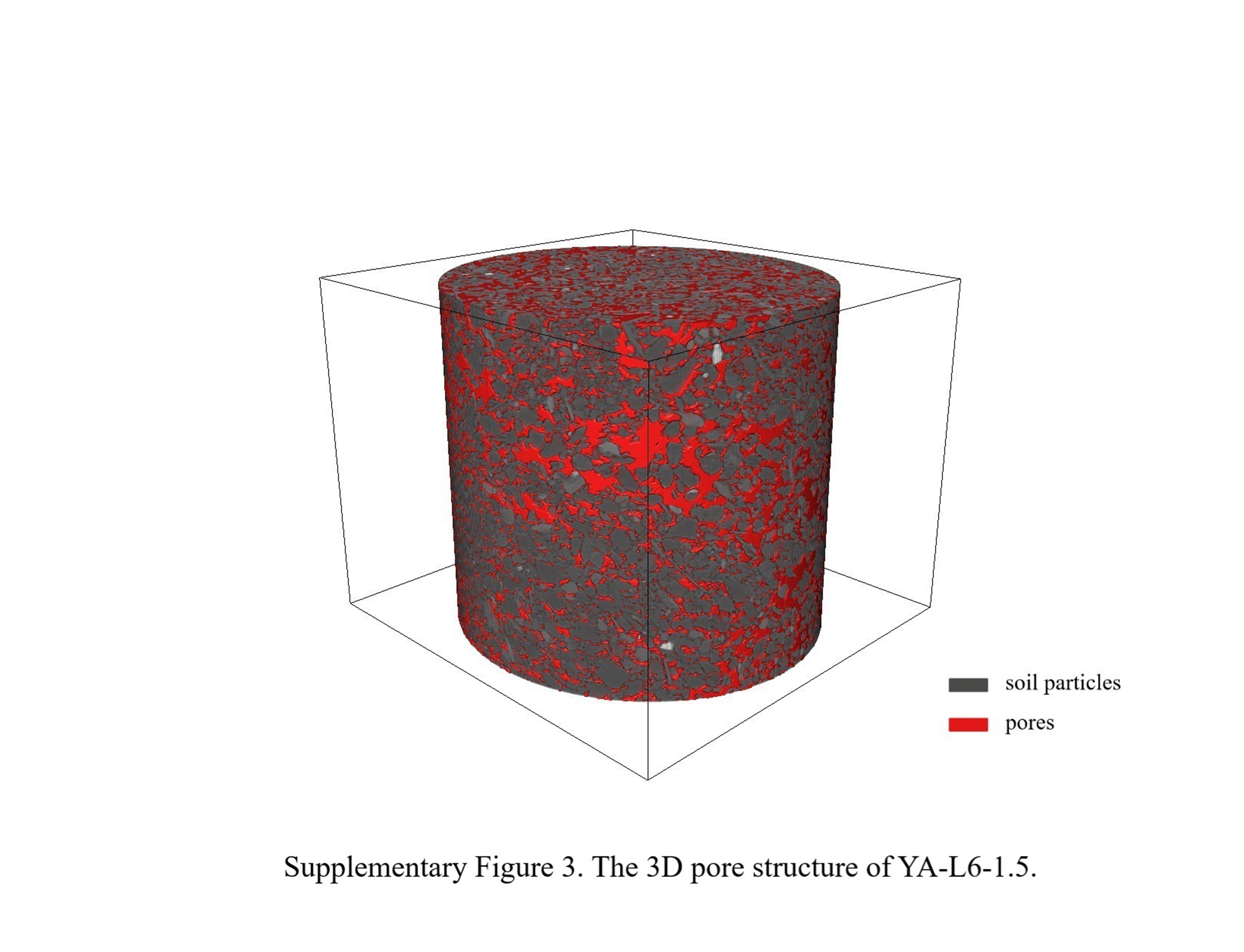

Supplement: Supplementary file 3 — Supplementary Figure S3. [file 41598_2020_65302_MOESM3_ESM.jpg]

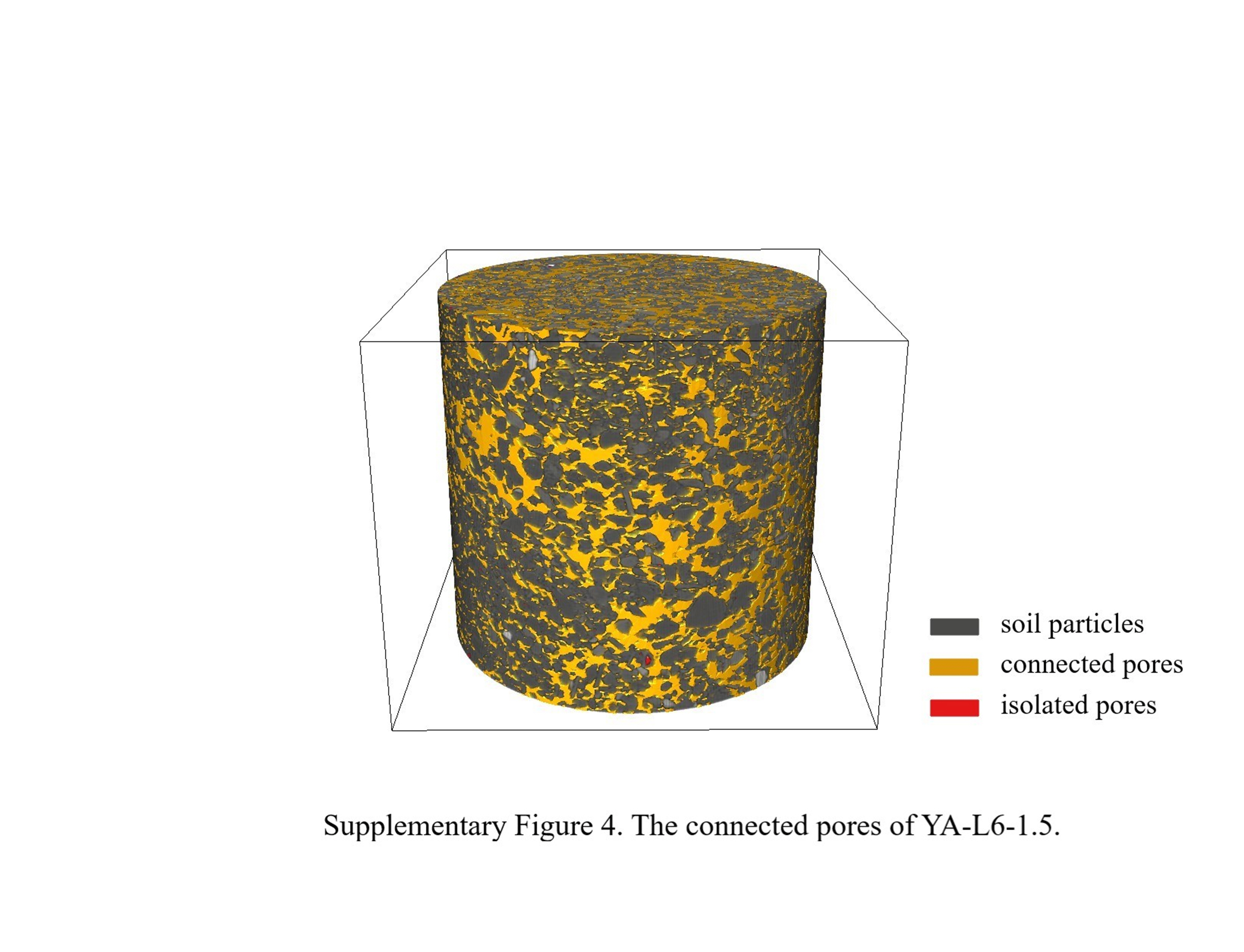

Supplement: Supplementary file 4 — Supplementary Figure S4. [file 41598_2020_65302_MOESM4_ESM.jpg]

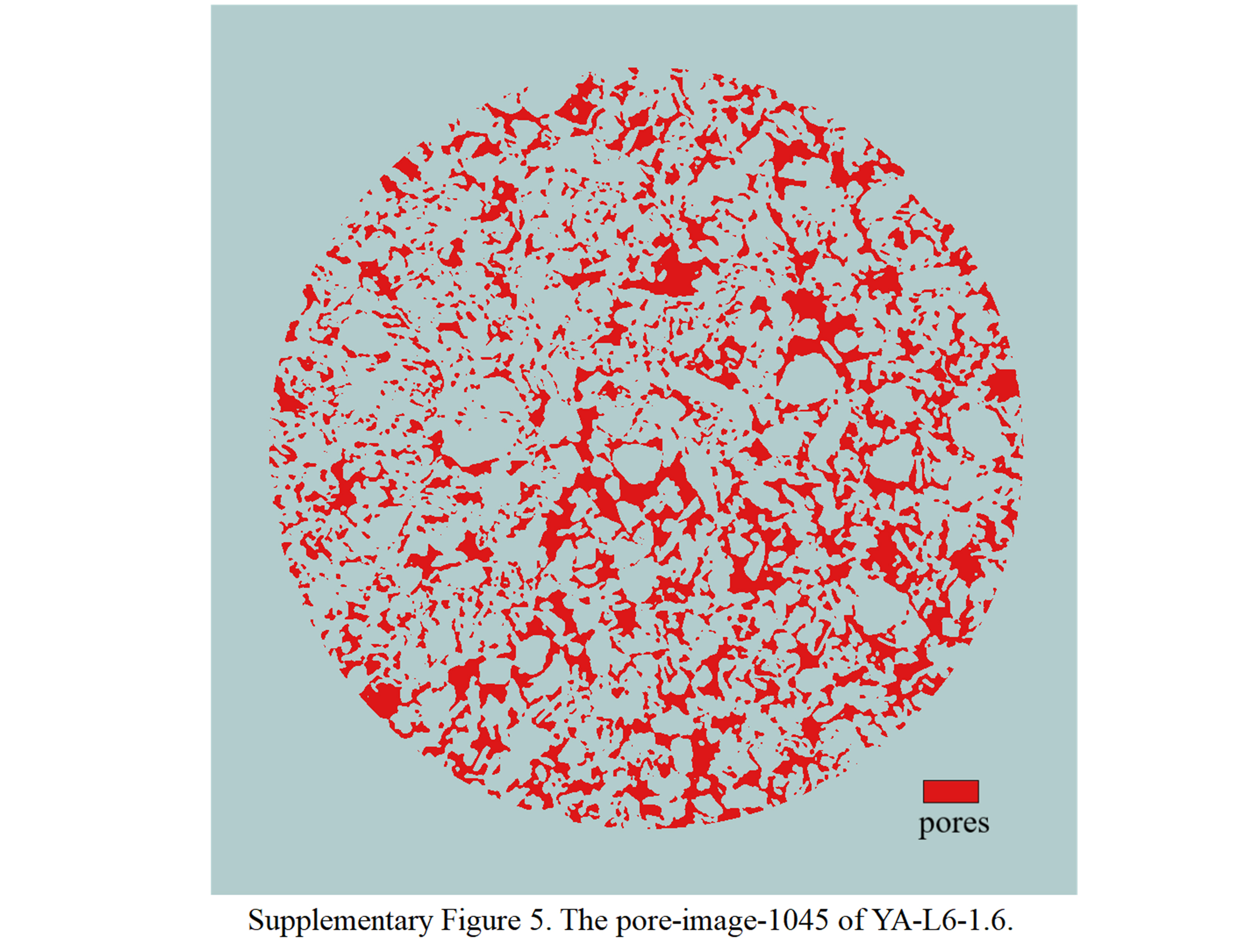

Supplement: Supplementary file 5 — Supplementary Figure S5. [file 41598_2020_65302_MOESM5_ESM.jpg]

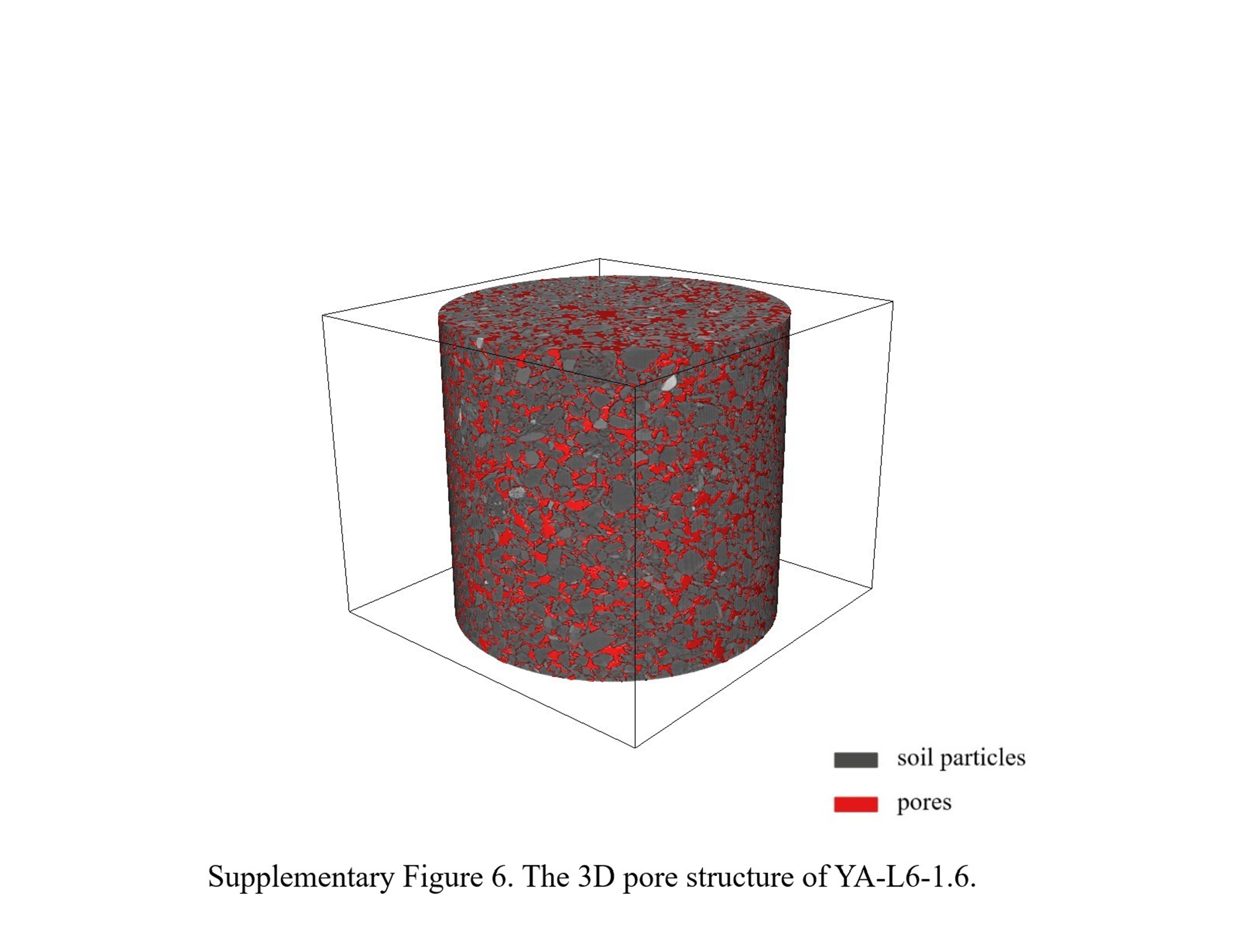

Supplement: Supplementary file 6 — Supplementary Figure S6. [file 41598_2020_65302_MOESM6_ESM.jpg]

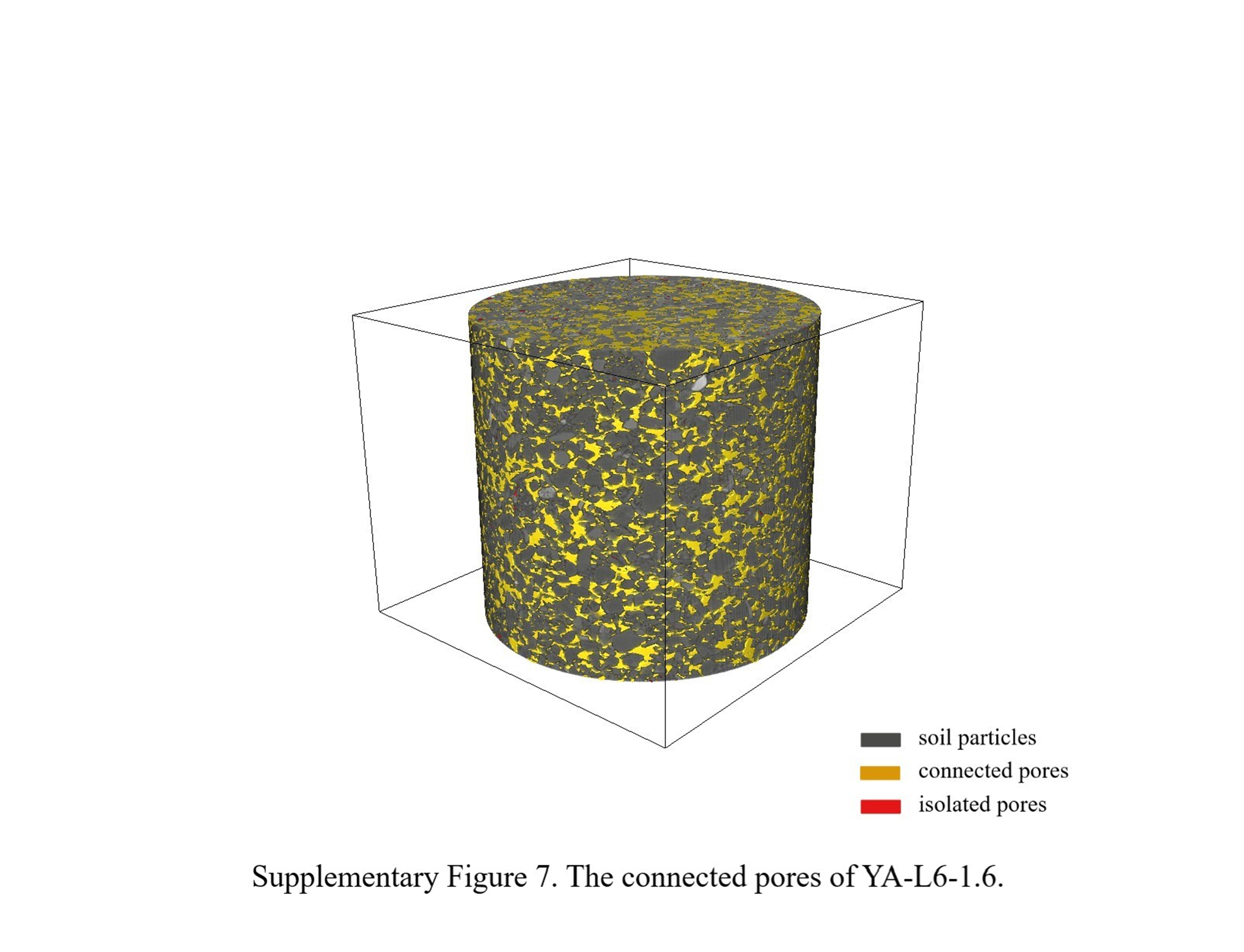

Supplement: Supplementary file 7 — Supplementary Figure S7. [file 41598_2020_65302_MOESM7_ESM.jpg]

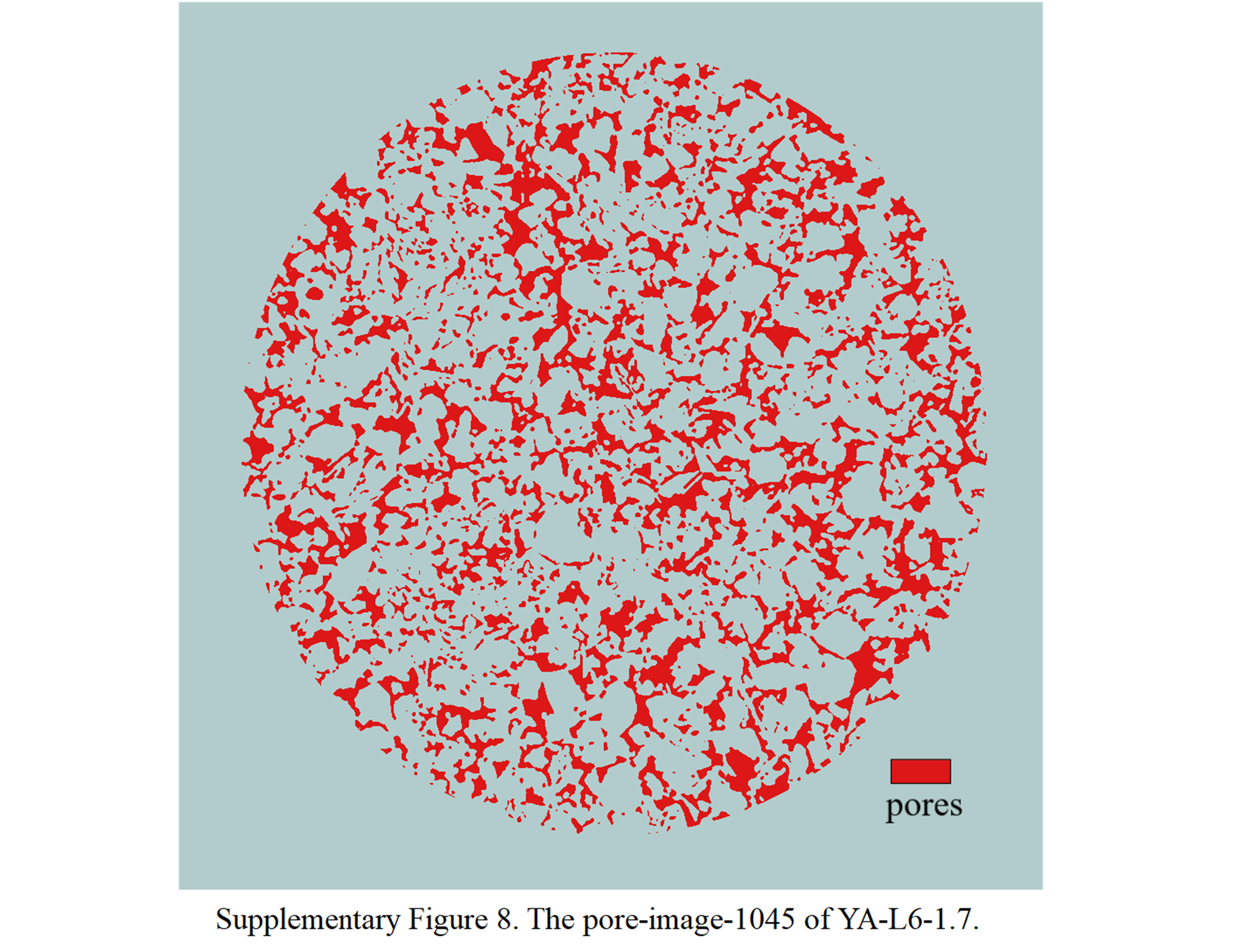

Supplement: Supplementary file 8 — Supplementary Figure S8. [file 41598_2020_65302_MOESM8_ESM.jpg]

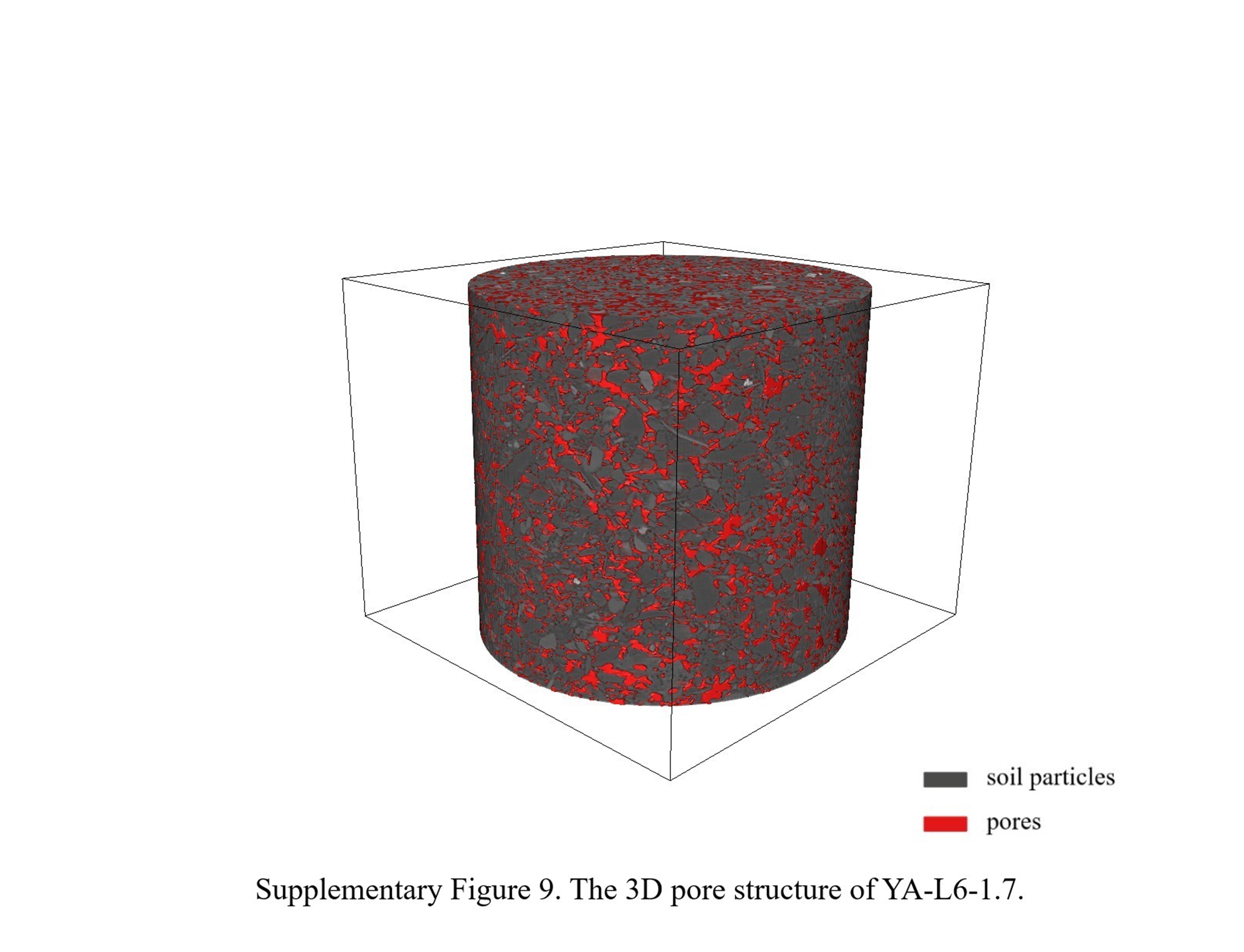

Supplement: Supplementary file 9 — Supplementary Figure S9. [file 41598_2020_65302_MOESM9_ESM.jpg]

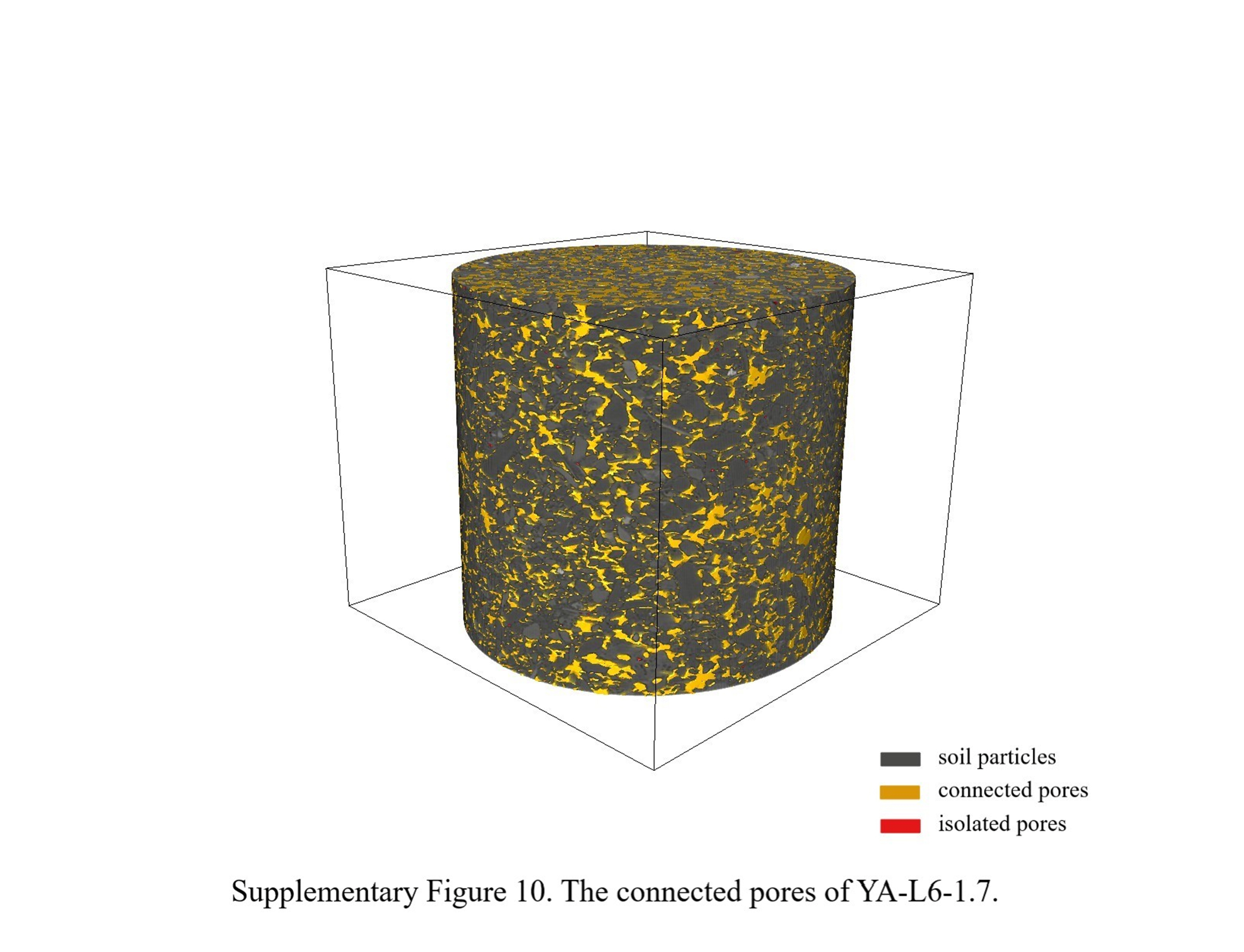

Supplement: Supplementary file 10 — Supplementary Figure S10. [file 41598_2020_65302_MOESM10_ESM.jpg]

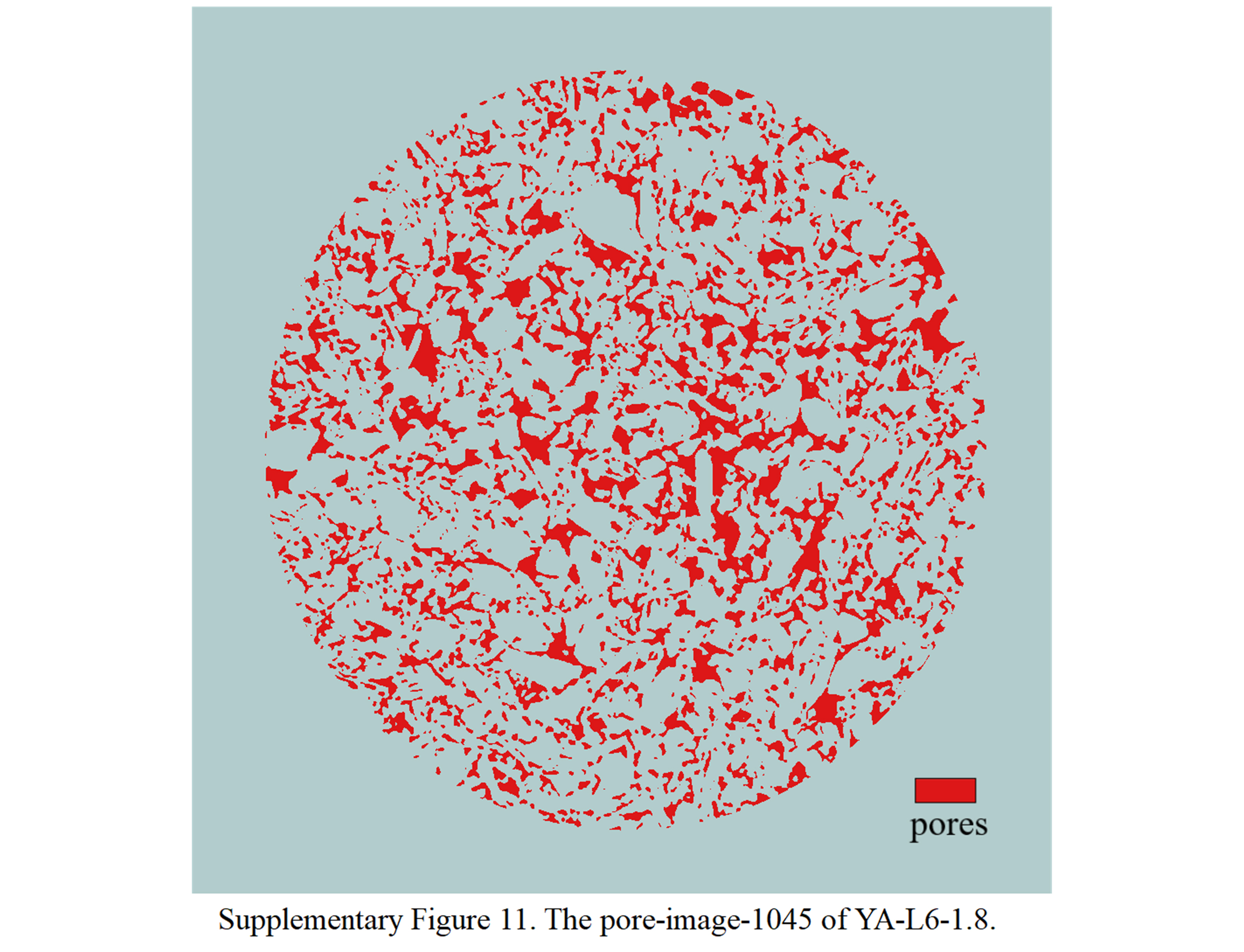

Supplement: Supplementary file 11 — Supplementary Figure S11. [file 41598_2020_65302_MOESM11_ESM.jpg]

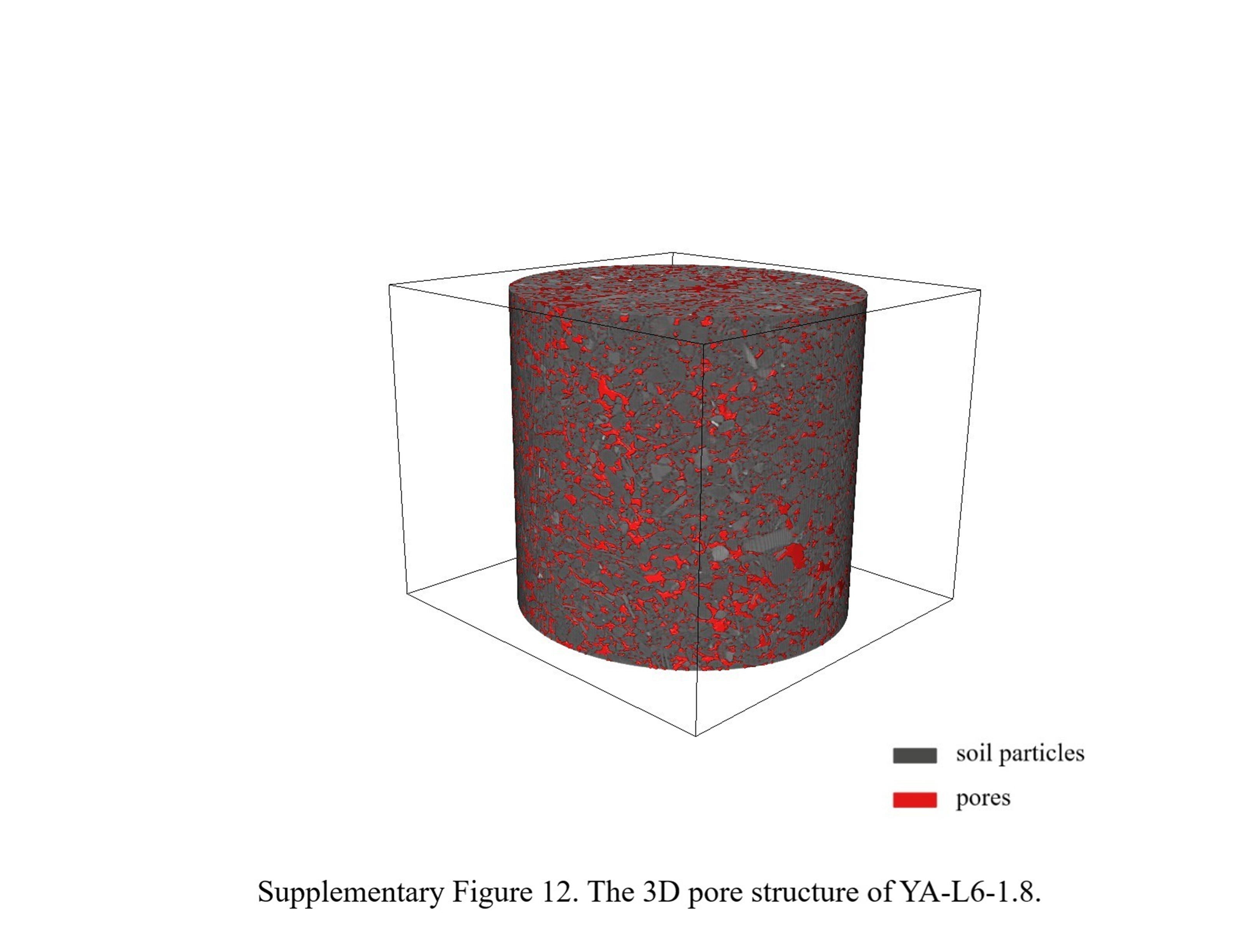

Supplement: Supplementary file 12 — Supplementary Figure S12. [file 41598_2020_65302_MOESM12_ESM.jpg]

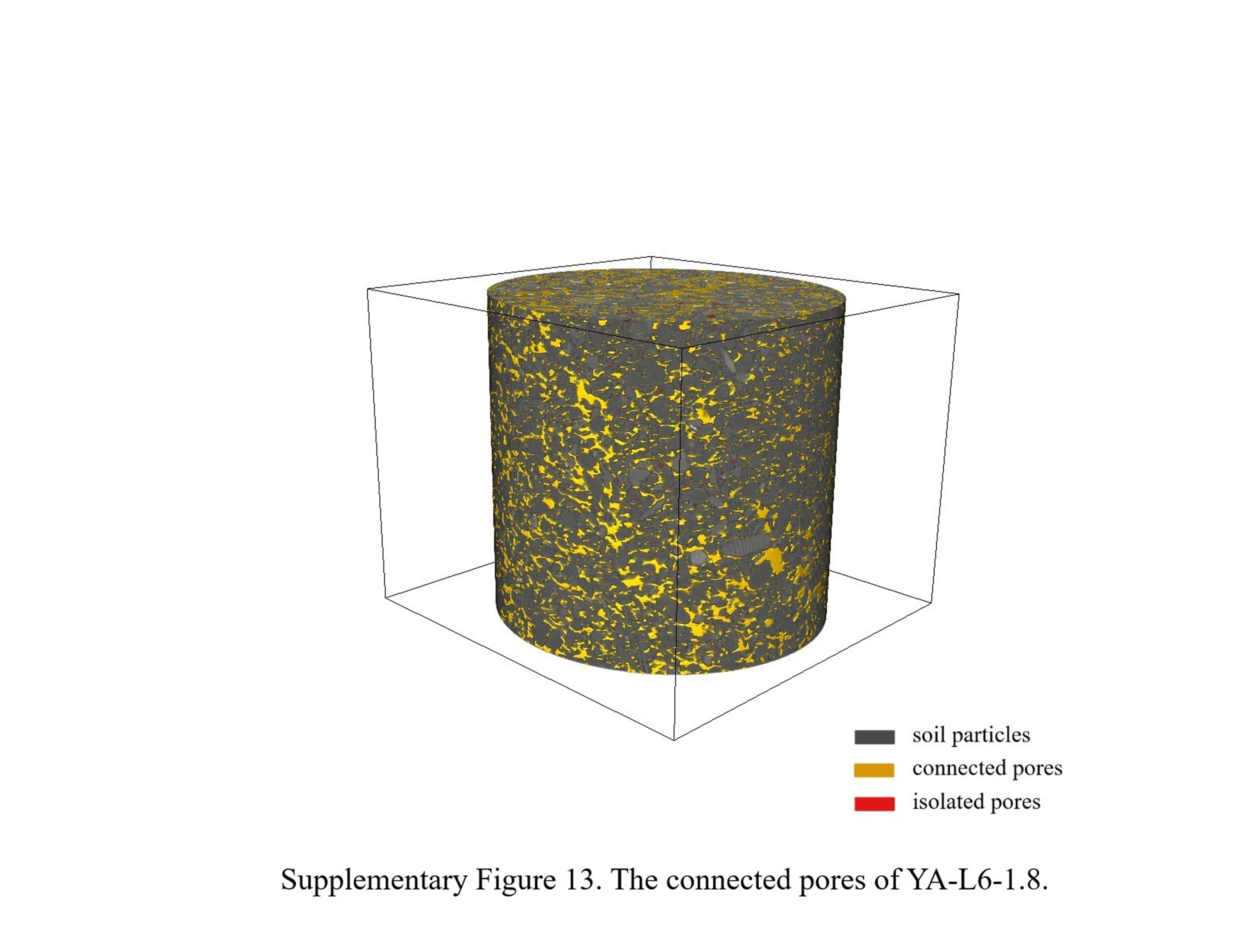

Supplement: Supplementary file 13 — Supplementary Figure S13. [file 41598_2020_65302_MOESM13_ESM.jpg]

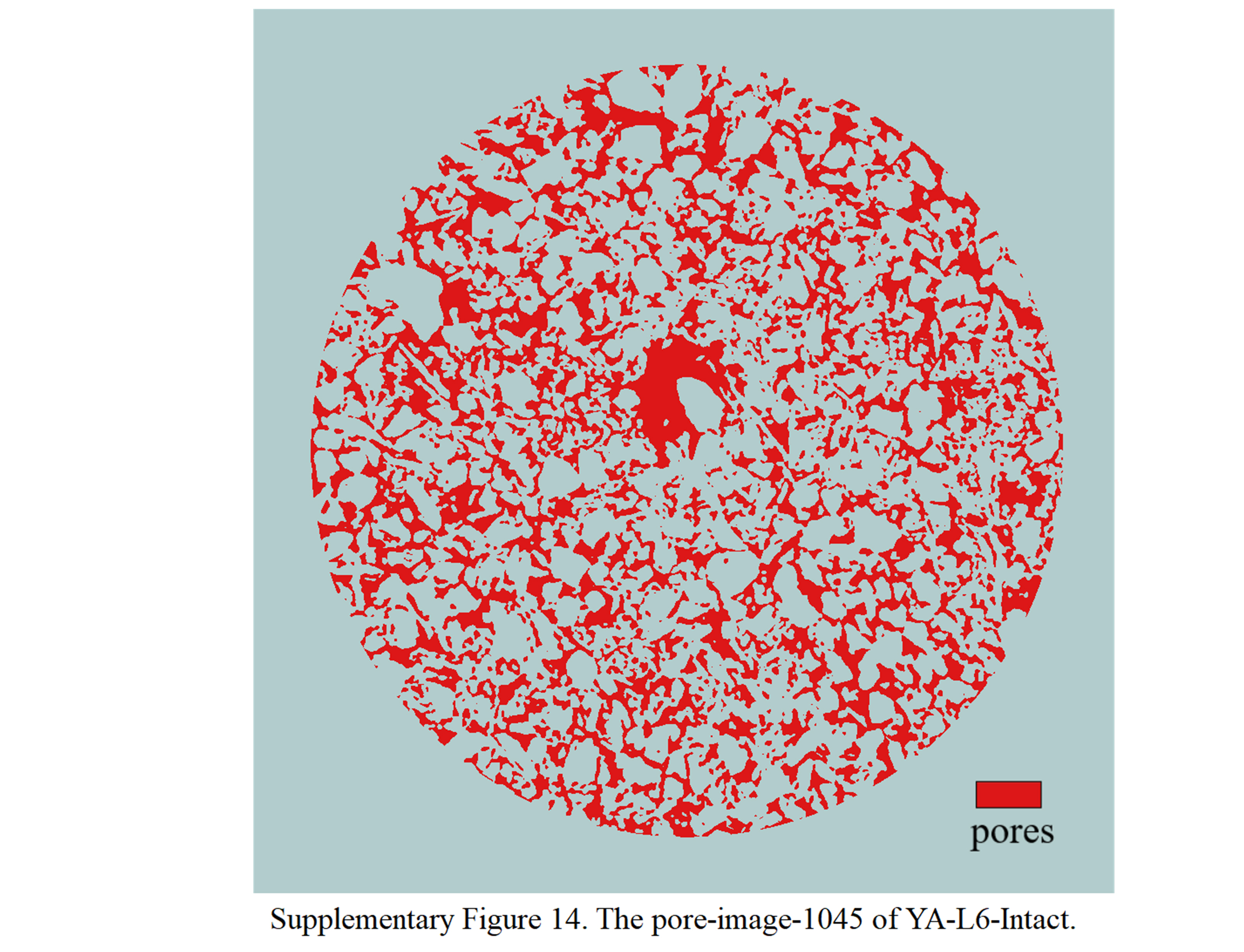

Supplement: Supplementary file 14 — Supplementary Figure S14. [file 41598_2020_65302_MOESM14_ESM.jpg]

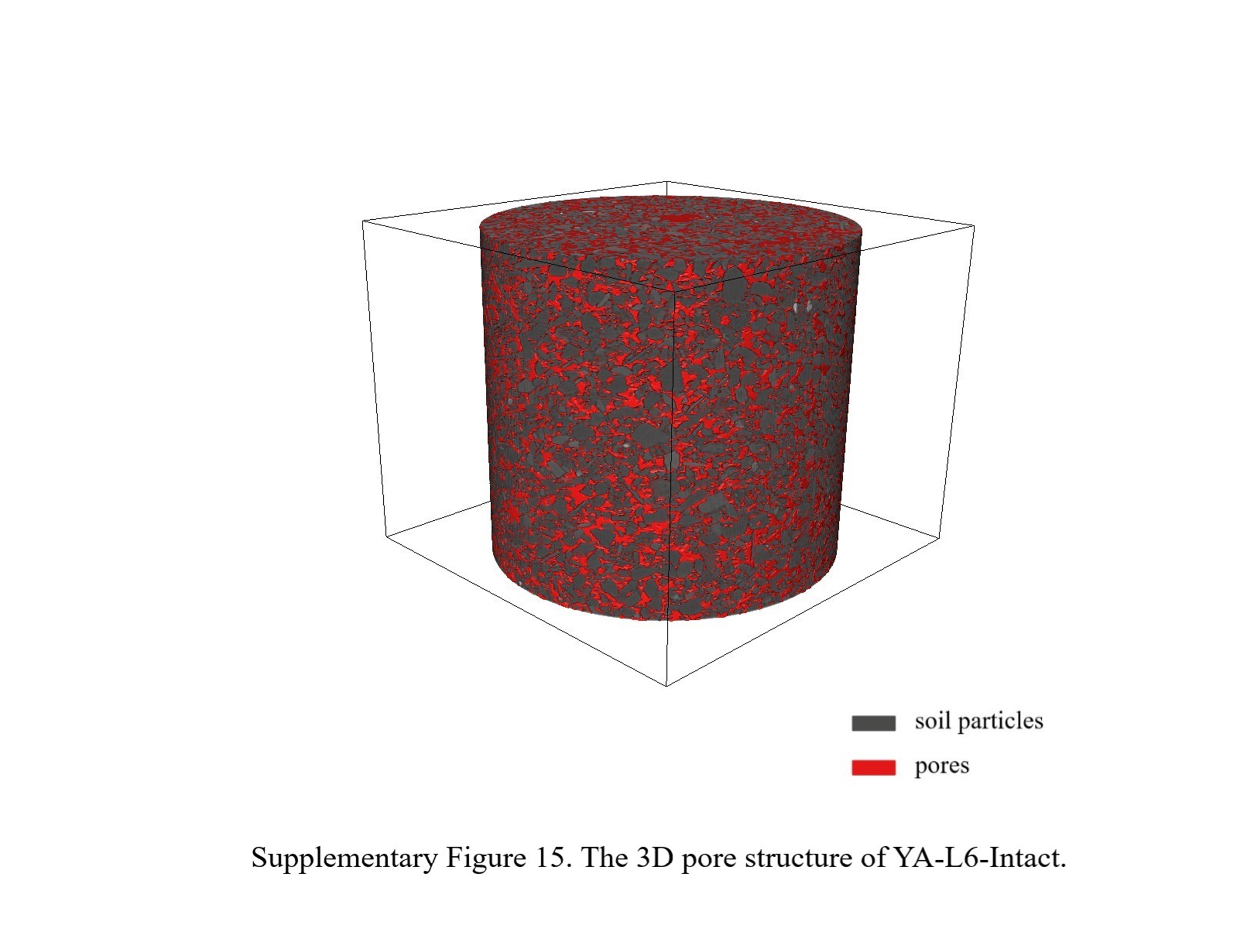

Supplement: Supplementary file 15 — Supplementary Figure S15. [file 41598_2020_65302_MOESM15_ESM.jpg]

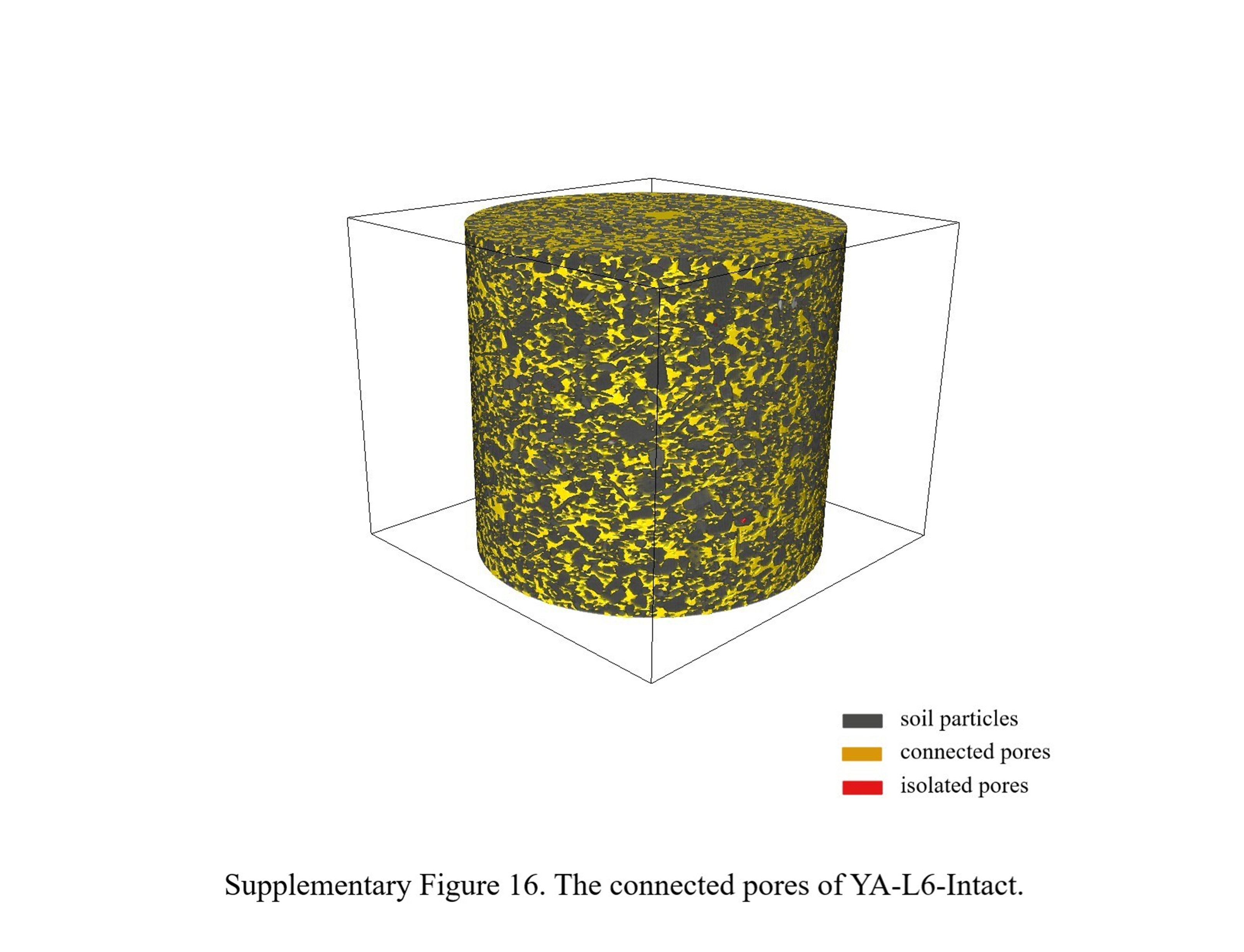

Supplement: Supplementary file 16 — Supplementary Figure S16. [file 41598_2020_65302_MOESM16_ESM.jpg]
